# Proteomic investigation of Cbl and Cbl-b in neuroblastoma cell differentiation highlights roles for SHP-2 and CDK16

**DOI:** 10.1016/j.isci.2021.102321

**Published:** 2021-03-17

**Authors:** Anna-Kathrine Pedersen, Anamarija Pfeiffer, Gopal Karemore, Vyacheslav Akimov, Dorte B. Bekker-Jensen, Blagoy Blagoev, Chiara Francavilla, Jesper V. Olsen

**Affiliations:** 1 Proteomics Program, Novo Nordisk Foundation Center for Protein Research, Faculty of Health and Medical Sciences, University of Copenhagen, Blegdamsvej 3B, 2200 Copenhagen, Denmark; 2 Department of Biochemistry and Molecular Biology, University of Southern Denmark, 5230 Odense, Denmark; 3 Division of Molecular and Cellular Functions, School of Biological Sciences, Faculty of Biology, Medicine and Health, University of Manchester, Manchester M13 9PL, UK

**Keywords:** Cell Biology, Systems Biology, Proteomics

## Abstract

Neuroblastoma is a highly heterogeneous embryonal solid tumor of the sympathetic nervous system. As some tumors can be treated to undergo differentiation, investigating this process can guide differentiation-based therapies of neuroblastoma. Here, we studied the role of E3 ubiquitin ligases Cbl and Cbl-b in regulation of long-term signaling responses associated with extracellular signal-regulated kinase phosphorylation and neurite outgrowth, a morphological marker of neuroblastoma cell differentiation. Using quantitative mass spectrometry (MS)-based proteomics, we analyzed how the neuroblastoma cell line proteome, phosphoproteome, and ubiquitylome were affected by Cbl and Cbl-b depletion. To quantitatively assess neurite outgrowth, we developed a high-throughput microscopy assay that was applied in combination with inhibitor studies to pinpoint signaling underlying neurite outgrowth and to functionally validate proteins identified in the MS data sets. Using this combined approach, we identified a role for SHP-2 and CDK16 in Cbl/Cbl-b-dependent regulation of extracellular signal-regulated kinase phosphorylation and neurite outgrowth, highlighting their involvement in neuroblastoma cell differentiation.

## Highlights

• Multi-layered proteomics captures cellular changes induced by Cbl/Cbl-b depletion
• SHP-2 and CDK16 protein and phosphorylation levels increase upon Cbl/Cbl-b depletion
• SHP-2 and CDK16 regulate phospho-ERK and neurite outgrowth in neuroblastoma cells
• Inhibition of SHP-2 or CDK16 reverts Cbl/Cbl-b knockdown effects on differentiation

## Graphical abstract

**Figure:**
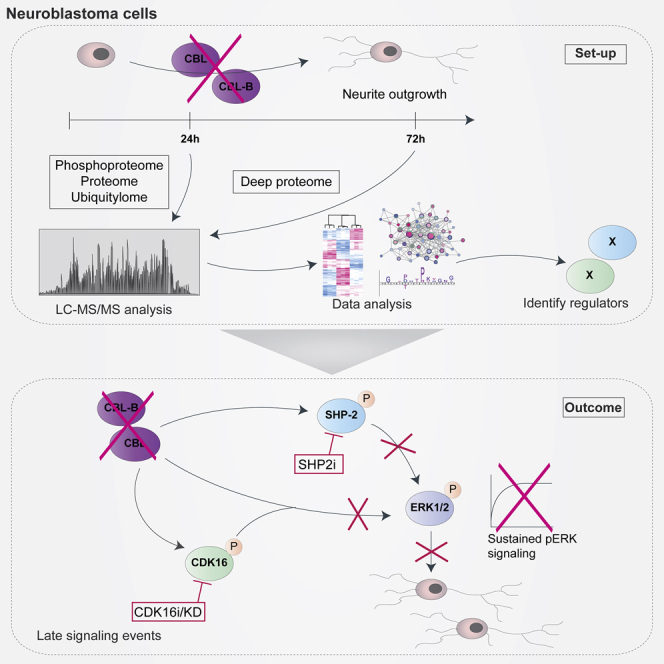

## Introduction

Neuroblastoma is the most common extracranial tumor in childhood and accounts for around 15% of pediatric cancer mortality (Johnsen et al., 2018; Matthay et al., 2016; Ratner et al., 2016). Neuroblastoma is an embryonal solid tumor arising in the developing sympathetic nervous system from cells of the neural crest, and tumor development is associated with failed neuronal differentiation. This disease is characterized by high heterogeneity, encompassing tumors with a high degree of variability in biological characteristics and clinical behaviors. Thus, tumors range from highly malignant and therapeutically resistant to tumors that spontaneously regress or differentiate (Ratner et al., 2016). Risk stratification (based on the International Neuroblastoma Risk Group [INRG] classification system) of patients for targeted therapy has reduced treatment load for low- and intermediate-risk patient groups for whom outcomes are excellent, while patients with high-risk disease are subjected to extensive treatment regimes. Yet, patient survival for high-risk neuroblastoma remains below 50% (Ambros et al., 2009; Cohn et al., 2009; Irwin and Park, 2015; Matthay et al., 2016). Survival is correlated with the level of tumor differentiation; thus, patients with more well-differentiated tumors have a more favorable outcome (Fredlund et al., 2008). Accordingly, differentiation therapy is an attractive strategy for neuroblastoma. Treatment with retinoic acid (RA) has been shown to induce neuroblastoma cell differentiation to a more mature neuronal state, and RA has become part of the standard treatment regime for patients with high-risk neuroblastoma (Johnsen et al., 2018; Matthay et al., 2009; Ratner et al., 2016). However, the exact mechanism of neuroblastoma differentiation remains an enigma, warranting further research and increased understanding of the signaling pathways underlying this process, as this can potentially lead to development of new and improved differentiation therapies and reveal new biomarkers.

The development of neuroblastoma can be seen as a consequence of failed neural crest cell differentiation. Different chemical agents and growth factors have been found to induce neuroblastoma cell differentiation *in vitro*, and receptor tyrosine kinase (RTK) signaling is involved in mediating this process (Edsjö et al., 2007). Neurotrophins and their cognate receptors, belonging to the tropomyosin-related kinase family of RTKs, have central roles in the development and maintenance of the nervous system, and neuroblastoma cells expressing tropomyosin-related kinase A (TrkA) have been shown to undergo differentiation in response to stimulation by its high-affinity ligand, nerve growth factor (NGF). Accordingly, high expression of TrkA is associated with low-stage tumors and a good prognosis (Brodeur and Bagatell, 2014; Edsjö et al., 2007; Mohlin et al., 2011). Signaling from RTKs is tightly regulated by a number of cellular mechanisms including ubiquitylation, internalization, and receptor degradation/recycling mediated by E3 ubiquitin ligases, such as the casitas B-lineage lymphoma protein, Cbl. The Cbl protein family consists of three members: c-Cbl (referred to as Cbl), Cbl-b, and Cbl-3. Cbl proteins are really interesting new gene (RING)-type E3 ubiquitin ligases, which can bind tyrosine-phosphorylated substrates through their tyrosine-kinase-binding domain to mediate ubiquitylation via an E2 ubiquitin-conjugating enzyme bound to the RING finger (Cooper et al., 2015; Thien and Langdon, 2001). Thus, Cbl proteins can specifically direct ubiquitylation of activated RTKs to negatively regulate signaling as it has been shown for a number of RTKs including epidermal growth factor receptor (EGFR), anaplastic lymphoma kinase (ALK), and TrkA (Ettenberg et al., 2001; Mazot et al., 2012; Soubeyran et al., 2002; Takahashi et al., 2011). In addition, the C-terminal regions of Cbl and Cbl-b contain phosphorylation sites and proline-rich regions, through which other adapter proteins can bind and thereby positively regulate signaling pathways by functioning as signaling scaffolds (Cooper et al., 2015; Thien and Langdon, 2001). We have previously identified a role for Cbl-b as a negative regulator of NGF-TrkA signaling in neuroblastoma cells and found that depletion of Cbl and Cbl-b was associated with induction of neurite outgrowth, a morphological marker of neuroblastoma differentiation (Emdal et al., 2015). The aim of the present study was to elucidate the general role of Cbl proteins in regulating RTK signaling and neurite outgrowth in neuroblastoma cells and to identify components of the signaling responses underlying these processes. We applied mass spectrometry (MS)-based quantitative proteomics to obtain an unbiased global view of Cbl-dependent cell signaling pathways in neuroblastoma cells. Analyzing multiple layers of signaling networks including the regulation of proteins and their post-translational modifications (PTMs) by MS-based quantitative proteomics can provide detailed information on cellular responses and reveal implicated molecular mechanisms (Emdal et al., 2018; Francavilla et al., 2013, 2016; Reckel et al., 2017). To gain insights into the roles of Cbl and Cbl-b in the regulation of neurite outgrowth, we investigated the global changes in the proteome, ubiquitylome, and phosphoproteome of Cbl protein-depleted neuroblastoma cells. This multi-layered data set revealed players involved in neuroblastoma cell differentiation and identified roles for signaling proteins insulin-like growth factor 1 receptor (IGF1R), SH2 domain-containing protein tyrosine phosphatase-2 (SHP-2) and cyclin-dependent kinase 16 (CDK16) in Cbl-dependent regulation of neuroblastoma cell function.

## Results

### E3 ubiquitin ligases Cbl and Cbl-b regulate neurite outgrowth in neuroblastoma cells in an ERK-dependent manner

**Figure 1:**
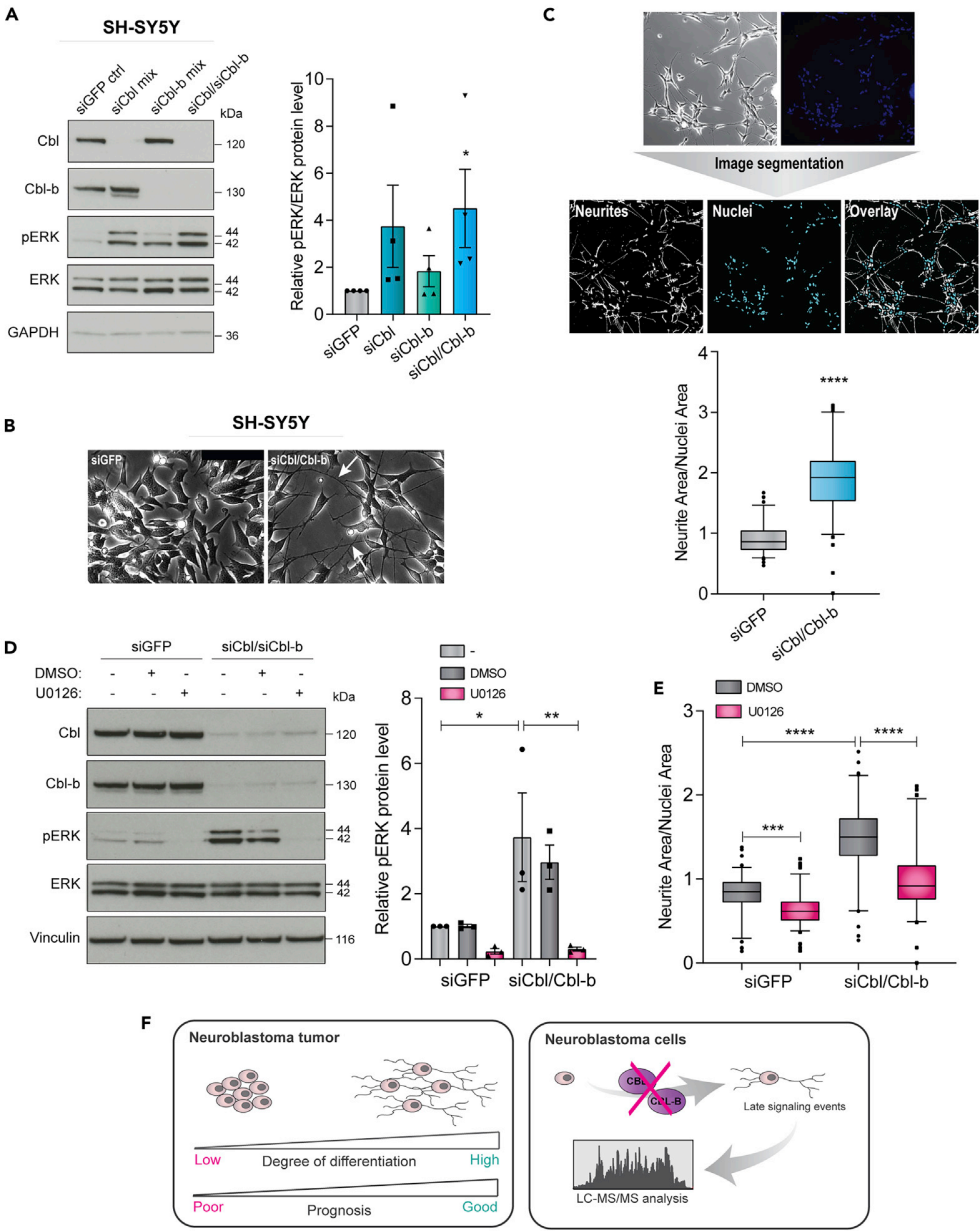
Cbl and Cbl-b regulate SH-SY5Y neuroblastoma cell differentiation by decreasing ERK phosphorylation levels and inhibiting neurite outgrowth (A–E) SH-SY5Y cells were treated with siRNA (as indicated) for 48-72 hr. (A) Lysates were subjected to immunoblotting (left panel) with antibodies against phospho-ERK/ERK or Cbl/Cbl-b. Right panel shows quantification relative to siGFP control (n = 4). (B) Representative images of siGFP and siCbl/Cbl-b-treated cells for neurite outgrowth (indicated by white arrows) visualization (scale bar, 50 μm). (C) Representative raw and segmented images of Cbl/Cbl-b knockdown SH-SY5Y cells. Segmented images (bottom panel) show neurites (white) and nuclei (cyan). Neurites and nuclei were quantified by “Neurite Outgrowth Quantification” software. (D) Immunoblotting (left panel) detecting indicated proteins in lysates of SH-SY5Y treated with Cbl/Cbl-b siRNA in conjunction with U0126 and quantification (right panel) relative to siGFP control. (E) Corresponding quantification of neurite outgrowth. (F) Graphical model of neuroblastoma differentiation and study design. Data in bar graphs are shown as means ± SEM and in boxplots as median with 95% confidence interval (CI) and representative of n = 3 independent experiments. ∗, ∗∗, ∗∗∗ and ∗∗∗∗ indicate p < 0.05, 0.01, 0.001, and <0.0001, respectively, compared to the corresponding control determined by one-sample t test (A), t test (C), or one-way ANOVA (D and E).

To study the roles of the closely related E3 ligases Cbl and Cbl-b in signaling and differentiation of neuroblastoma cells, we initially employed a panel of three neuroblastoma cell lines, namely SH-SY5Y, NB1, and IMR-32. These were selected based on their ability to form cellular projections referred to as neurite outgrowths, a morphological marker of cell differentiation, in response to different stimuli (Halder et al., 2015; Nishimura et al., 1995; Påhlman et al., 1995; Rettig et al., 2015). Furthermore, these cell lines express different combinations of oncogenes known to be important for neuroblastoma development and progression, such as amplified *MYCN* and mutated or amplified ALK (Cheung and Dyer, 2013; Emdal et al., 2018; Matthay et al., 2016) (Figure S1A). To disrupt the function of Cbl and/or Cbl-b, we used small interfering RNA (siRNA)-based knockdown and found by microscopy-based visual inspection that depletion of both induced increased neurite outgrowth in all cell lines, however, less evident in IMR-32. In addition to this phenotypic change, we saw a concomitant increase in extracellular signal-regulated kinase (ERK) phosphorylation upon knockdown of Cbl E3 ligases (Figures 1A, 1B, and S1B–S1E). This is in accordance with the important role of activated ERK in cellular differentiation and neurite outgrowth and supports previous findings (Emdal et al., 2015; Grewal et al., 1999; Robinson et al., 1998). Thus, we applied neurite outgrowth in combination with increased levels of ERK phosphorylation as a proxy for neuroblastoma cell differentiation throughout this study. Importantly, we found that the magnitude and robustness of the differentiation response was increased by simultaneous depletion of Cbl and Cbl-b compared to depletion of individual proteins (Figures 1A, 1B, and S1B–S1E). The magnitude of the response varied between cell lines, with IMR-32 being the least responsive to Cbl/Cbl-b depletion (Figures S1C and S1E) and SH-SY5Y cells displaying the largest increase in ERK phosphorylation levels, as well as the most obvious change in morphological phenotype. Thus, we chose SH-SY5Y as the model system for further analysis of the role of Cbl/Cbl-b in neuroblastoma cell differentiation. To validate the specificity of the effects observed upon Cbl/Cbl-b knockdown, we depleted both Cbl and Cbl-b individually in SH-SY5Y using three different siRNAs (S1, S2, and S3) against each protein, as well as two different pools (pooling either S1 or S3) to deplete Cbl and Cbl-b simultaneously. Overall, these results confirmed, for all tested sequences, that knockdown of Cbl proteins leads to increased ERK phosphorylation and increased neurite outgrowth and that this response is amplified by dual depletion of both Cbl and Cbl-b (Figures S1F and S1G). Since RA is an established treatment used for differentiation therapy of patients with neuroblastoma (Matthay et al., 2009), we applied RA treatment as a positive control to compare with our experimental approach. Accordingly, RA treatment over a time course of 24-120 hr caused increased ERK phosphorylation and simultaneously enhanced neurite outgrowth (Figures S1H and S1I), thus validating our system and approach to study neuroblastoma differentiation (Påhlman et al., 1984).

To accurately measure and quantify neurite outgrowth in an unbiased manner from a large number of cells across conditions, we utilized a ScanR fully automated screening microscope and developed a MATLAB-based “Neurite Outgrowth Quantification” software tool (see workflow; Figure S1J). To avoid harsh treatments of the cells, we developed the tool for live-cell imaging and used Hoechst staining to visualize nuclei. Figure 1C depicts a comparison between raw and segmented images. Using this setup, we validated the visually observed increase in neurite outgrowth induced by Cbl/Cbl-b depletion and RA treatment in SH-SY5Y in an unbiased and quantifiable manner (Figures 1C and S1K).

To assess whether ERK activity played an essential role in the differentiation of the SH-SY5Y cells induced by Cbl/Cbl-b depletion, we combined siRNA-mediated knockdown with inhibition of mitogen-activated protein kinase kinase (MEK) by using the small molecule inhibitor U0126. We found that inhibiting MEK activation abolished ERK phosphorylation (Figure 1D) and indeed, inhibition of ERK activation, correlated with a significant decrease in knockdown-induced neurite outgrowth (Figure 1E). Thus, mitogen-activated protein kinase (MAPK) cascade activity is crucial for the induction of neurite outgrowth upon Cbl/Cbl-b depletion, further validating our approach to use phospho-ERK as a marker of neuroblastoma cell differentiation. Accordingly, we applied this model system combined with MS-based proteomics screens to study Cbl-dependent signaling pathways underlying neuroblastoma cell differentiation (Figure 1F).

### Proteome changes link Cbl proteins to regulation of long-term cellular responses

**Figure 2:**
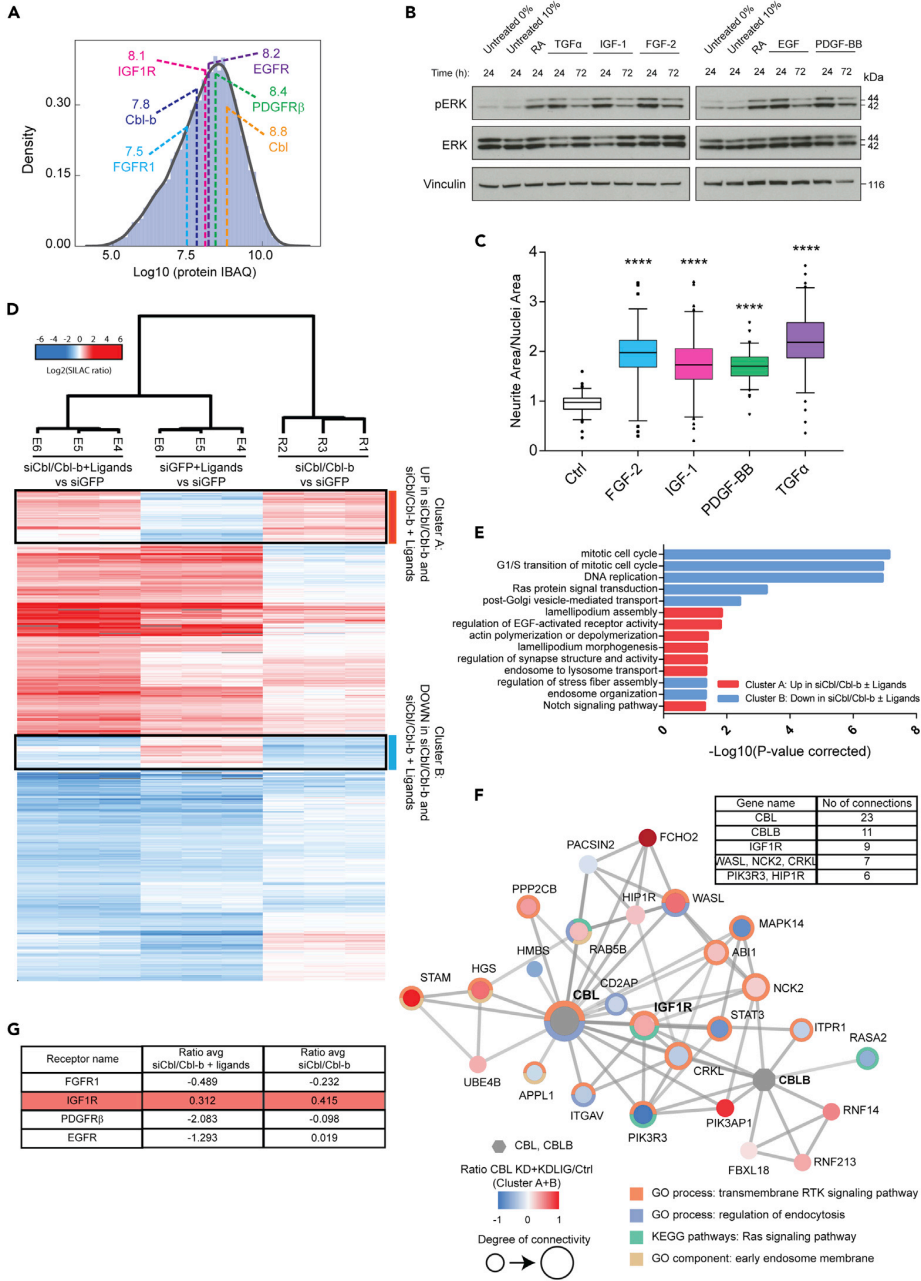
Deep proteome analysis to study the impact of Cbl/Cbl-b on long-term RTK signaling responses in SH-SY5Y cells (A) Density analysis of protein abundances (IBAQ) in SH-SY5Y cells highlighting Cbl/Cbl-b and RTKs, shown with their corresponding IBAQ values (Log_10_). (B) Immunoblotting for phospho-ERK of lysates from SH-SY5Y cells stimulated with FGF-2, IGF-1, PDGF-BB, or TGFα (or EGF). RA was used as a positive control. (C) Neurite outgrowth quantification of cells exposed to the same conditions as in (B). Data are shown as medians with 95% CI. ∗∗∗∗ indicates p < 0.0001 (one-way ANOVA) compared to the control. Data are representative of n = 3 independent experiments. (D) Hierarchical clustering of proteins with significantly regulated abundance (ANOVA) in response to treatment with Cbl/Cbl-b siRNA (72 hr) and/or RTK ligand cocktail (FGF-2, IGF-1, PDGF-BB, and TGFα; 48 hr). The two clusters selected for further analyses are highlighted. Data are presented with log_2_ SILAC ratios all relative to siGFP control. (E) Bar graph illustrating significantly overrepresented GO terms for biological process (GOBP) for cluster A (red) and B (blue). (F) Functional network analysis of proteins in clusters A and B, displaying first degree connections of Cbl and Cbl-b (gray). Node color indicates regulation on protein level and surrounding colored circles indicate enriched GO terms and KEGG pathways (see legend). The table summarizes the number of connections for the most connected proteins. (G) Table summarizing average ratios from the proteome data (relative to the siGFP control) for the RTK panel.

The Cbl protein family is known to modulate the signaling response of several RTKs through regulation of RTK internalization and degradation (Mazot et al., 2012; Mohapatra et al., 2013; Rorsman et al., 2016; Shtiegman et al., 2007). Thus, we hypothesized that the effect of Cbl/Cbl-b depletion on ERK phosphorylation and neurite outgrowth may involve altered expression or activity of one or more RTKs. In support of this hypothesis, activation of several different RTKs has previously been shown to induce neurite outgrowth of neuroblastoma cell lines (Lavenius et al., 1994; Motegi et al., 2004). Initially, we identified a panel of prospective RTK candidates in our SH-SY5Y cells by analyzing the distribution of protein abundances in a previously published deep proteome (Bekker-Jensen et al., 2017) (Figure 2A). We selected EGFR, IGF1R, platelet-derived growth factor receptor β (PDGFRβ), and fibroblast growth factor receptor 1 (FGFR1) as RTKs with differential expression levels and stimulated the cells with their respective ligands. Indeed, treatment with FGF-2, IGF-1, PDGF-BB, and transforming growth factor α (TGFα)/EGF, respectively, induced ERK phosphorylation and increased neurite outgrowth upon long-term stimulation over 24–72 hr (Figures 2B and 2C). To identify protein abundance changes associated with Cbl/Cbl-b proteins and neurite outgrowth induction, we performed a large-scale quantitative MS-based proteome analysis of SH-SY5Y cells after 72 hr of Cbl/Cbl-b depletion (Figure S2A). To assess changes in protein levels under both basal as well as conditions of increased RTK activation, we included siGFP control and siCbl/Cbl-b conditions stimulated with a “ligand cocktail” mixture of RTK ligands FGF-2, IGF-1, PDGF-BB, and TGFα. For quantitative comparison between conditions, we applied a double and triple SILAC (stable isotope labeling by amino acids in cell culture)-based setup combined with nanoflow high-performance liquid chromatography-tandem mass spectrometry (LC-MS/MS) and analyzed each experiment in triplicates (Figure S2A). Using our established deep proteome approach (Bekker-Jensen et al., 2017), we identified and quantified 10,030 proteins with Pearson correlation coefficients of 0.60–0.84 between SILAC replicates of the same condition (Figures S2A–S2C and Table S1). We performed an analysis of variance (ANOVA) statistical test and identified 2980 proteins with significantly changed abundance between the analyzed conditions. Unsupervised hierarchical clustering of the significantly regulated proteins resulted in grouping according to treatment and separated the proteins into six major clusters. To decipher the role of Cbl and Cbl-b, we focused on the proteins specifically upregulated or downregulated for the siCbl/Cbl-b vs. siGFP condition both in the presence and absence of ligand stimulation as represented in cluster A (up for siCbl/Cbl-b and siCbl/Cbl-b + ligands) and cluster B (down in siCbl/Cbl-b and siCbl/Cbl-b + ligands) (Figures 2D and S2B and Table S1). Cbl and Cbl-b displayed the lowest SILAC ratio among the downregulated proteins found in cluster B, serving as a quality control of our knockdown experiment. Gene ontology (GO) enrichment analysis for biological processes revealed that upregulated proteins were associated with functions related to RTK signaling regulation, endosomal transport, and cytoskeletal rearrangements (underlying processes like neurite outgrowth) (Figure 2E). The downregulated proteins were mostly associated with cell cycle and DNA replication, which could correlate with the fact that differentiation is often associated with decreased cell cycle progression (Chu and Cheung, 2003; Veal et al., 2002) (Figure 2E).

A STRING-based functional network of proteins in clusters A and B directly linked to Cbl and Cbl-b revealed that our analysis identified several players connected to Cbl protein biology including IGF1R, which was one of the upregulated proteins most strongly connected to Cbl and Cbl-b (Figure 2F). Notably, IGF1R was identified as the only RTK displaying an upregulated expression level in response to Cbl/Cbl-b depletion, independently of receptor stimulation by exogenously added ligands, as shown in Figure 2G. Thus, we considered that downregulation of IGF1R by Cbl/Cbl-b might contribute to the inhibitory effect on neurite outgrowth. Supporting this concept, we observed that increased activity of IGF1R can induce neurite outgrowth of SH-SY5Y associated with increased phosphorylation of ERK (Figures 2B and 2C), which is in line with previous reports (Kim et al., 1997).

### Analysis of global changes in ubiquitylation upon Cbl protein depletion

Cbl and Cbl-b function as E3 ubiquitin ligases, and a major function of ubiquitylation is regulating protein stability and degradation. Thus, we investigated global changes in ubiquitylation events upon depletion of Cbl and Cbl-b and how these correlated with changes in the proteome. We analyzed the ubiquitylome of siCbl/Cbl-b-treated SH-SY5Y cells using a double SILAC-based setup (Figure S3A), with antibody-based enrichment of ubiquitylated peptides performed using UbiSite, which allowed us to strictly enrich for ubiquitylated peptides (Akimov et al., 2018). We identified and quantified 6546 ubiquitylated sites distributed on 2486 proteins. Using a one-sample t test, we found 1560 sites to be significantly regulated, with 437 sites being downregulated by Cbl/Cbl-b knockdown and 1123 being upregulated (Table S2). To assess how changes in ubiquitylation correlated with altered protein expression, we plotted the SILAC ratios of all the 1560 regulated ubiquitylated sites against the SILAC ratio of their corresponding protein as found in the 72 hr proteome (Figure S3B, Tables S1 and S2). In addition, we identified 76 shared proteins regulated by siCbl/Cbl-b treatment both on the level of ubiquitylation and in the 72 hr proteome (Figure S3C). Functional network analysis of these 76 proteins revealed several proteins connected to Cbl and Cbl-b displaying different levels of ubiquitylation and protein abundance regulation with only 18 displaying an inverse correlation (Figure S3D). This suggests that the changes in ubiquitylation observed upon Cbl/Cbl-b depletion are implicated in regulation of cell processes in a more complex fashion than simply by altering protein abundance.

### Cbl proteins regulate RTK levels and ERK activation in neuroblastoma cells

**Figure 3:**
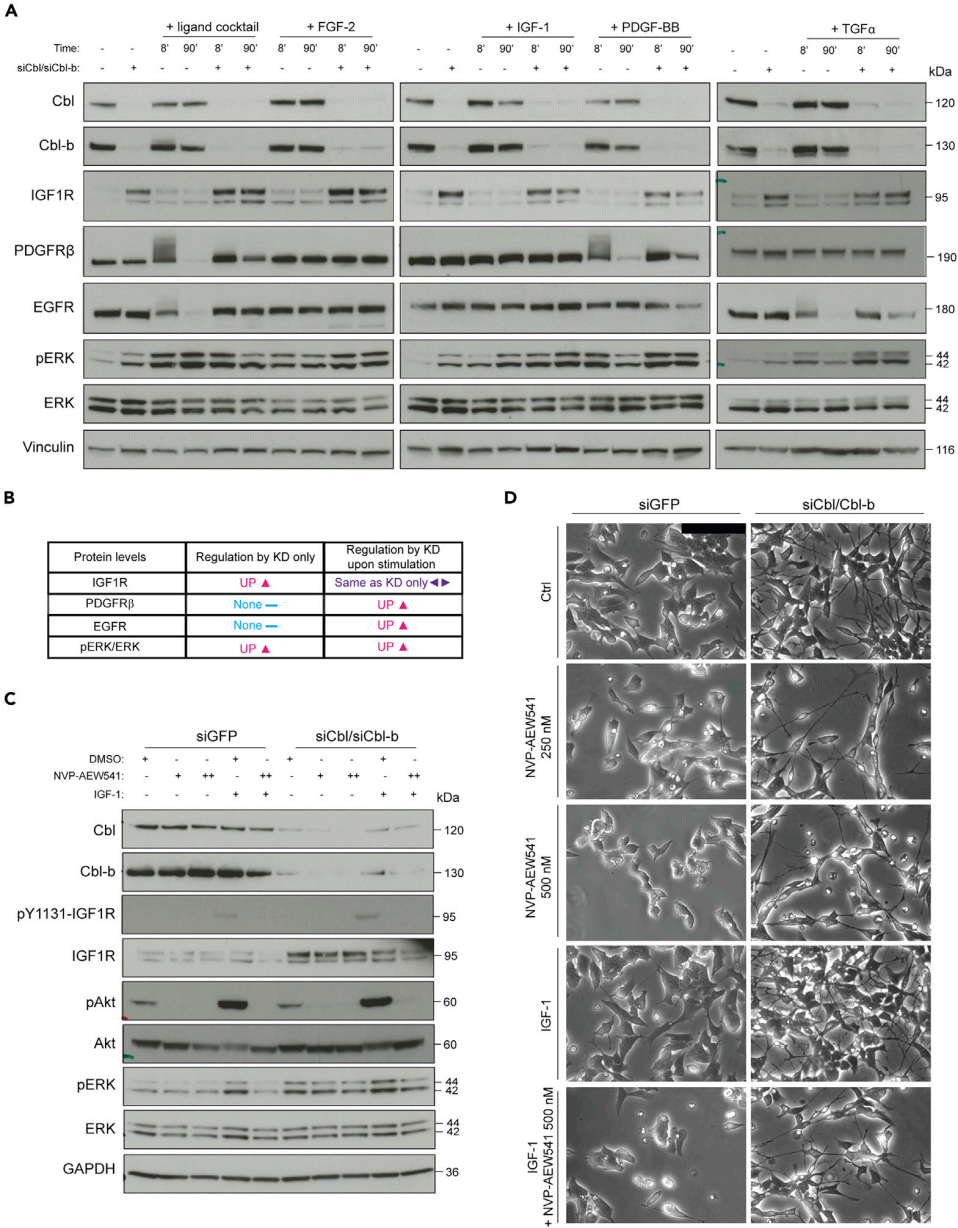
Cbl and Cbl-b regulate MAPK signaling (A and B) SH-SY5Y cells were treated with control siRNA or siRNAs targeting Cbl and Cbl-b for 48 hr prior to stimulation of RTKs with FGF-2, IGF-1, PDGF-BB, or TGF-α as single ligands or a mix (ligand cocktail) for 8 or 90 min. Lysates were immunoblotted using the indicated antibodies as shown in the representative blots (A) and with findings summarized in table (B). (C) Immunoblotting of lysates from SH-SY5Y cells treated with siGFP or siCbl/Cbl-b and IGF1R inhibitor (NVP-AEW541; + and ++ indicates 250 or 500 nM, respectively) for 72 hr, detecting the indicated proteins. (D) Representative images based on the same conditions as in (C) (scale bar, 50 μm). Data are representative of three independent experiments (n = 3).

We validated the changes in protein abundance of the identified RTKs by western blotting and investigated whether potential changes in expression levels correlated with altered magnitude of signaling responses (e.g. ERK phosphorylation) upon short-term stimulation. Control or Cbl/Cbl-b knockdown cells were stimulated with either the ligand cocktail or FGF-2, IGF-1, PDGF-BB, or TGFα, individually, for 8 and 90 min, respectively. These time points served as a proxy for “maximum” receptor activation (8 min) and receptor internalization/degradation (90 min) (Francavilla et al., 2016). In agreement with the proteome data, results showed that IGF1R protein levels were increased upon depletion of Cbl/Cbl-b compared to siGFP control cells independent of ligand stimulation (Figures 3A and 3B). The levels of both PDGFRβ and EGFR increased in Cbl/Cbl-b knockdown cells relative to control after 90 min of receptor stimulation, whereas basal levels were not affected by Cbl/Cbl-b depletion. Correspondingly, levels of receptor ubiquitylation, as indicated by the smeared staining band were apparently decreased after 8 min of stimulation in knockdown cells (Figures 3A and 3B). Changes in receptor levels were persistently associated with similar differences in downstream signaling events as exemplified here by increased levels of phosphorylated ERK, a common converging signaling hub for activated RTKs (Figures 3A and 3B). Furthermore, ERK phosphorylation revealed distinct kinetics of the activated RTKs in these cells. For example, ERK phosphorylation downstream of exogenously activated IGF1R peaked at 90, compared to 8 min under both control and Cbl/Cbl-b knockdown conditions, whereas phospho-ERK levels were dramatically decreased in the presence of Cbl/Cbl-b after 90 min of PDGFR and EGFR activation. These findings together with the proteome data support our hypothesis that IGF1R could be the central RTK implicated in the long-term signaling responses regulating neurite outgrowth and ERK phosphorylation in the SH-SY5Y cells. To further investigate the functional role of IGF1R, we treated the cells with siRNA against Cbl and Cbl-b, while simultaneously inhibiting IGF1R activity using either linsitinib (OSI-906) or NVP-AEW541. While NVP-AEW541 is not approved for clinical use, linsitinib has been in 24 clinical trials; however, trials were discontinued due to suboptimal results. Both inhibitors specifically inhibited IGF1R phosphorylation in response to IGF-1 and abolished Akt phosphorylation, a central mediator of IGF1R signaling, both in stimulated as well as basal conditions. This points to a central role of IGF1R in regulation of Akt signaling in the neuroblastoma cells. Furthermore, inhibitor treatment decreased IGF-1-induced ERK phosphorylation. However, in contrast to our hypothesis, the increase in ERK phosphorylation observed in response to depletion of Cbl and Cbl-b was not noticeably affected by IGF1R inhibition (Figures 3C and S3E). Accordingly, manual visual inspection of cell morphology by phase-contrast microscopy revealed that neurite outgrowth induced by Cbl/Cbl-b depletion was not dependent on IGF1R activity. However, an apparent decrease in cell numbers upon treatment with IGF1R inhibitor, under all conditions including knockdown, was evident despite the sustained ERK activation (Figures 3D and S3F). These findings suggest that IGF1R signaling induces a mitogenic rather than a cell differentiation response in this context. To investigate this, we treated Cbl/Cbl-b knockdown or siGFP control SH-SY5Y cells with increasing doses of the IGF1R inhibitor OSI-906 and performed cell survival assays. These data showed that IGF1R signaling seems to support neuroblastoma cell survival and that Cbl/Cbl-b-knockdown cells were indeed slightly more sensitive toward IGF1R inhibition than control cells, displaying a lower OSI-906 IC50 value (Figure S3G).

### Late phospho-signaling analysis reveals potential regulators of the neurite outgrowth response induced by Cbl protein depletion

**Figure 4:**
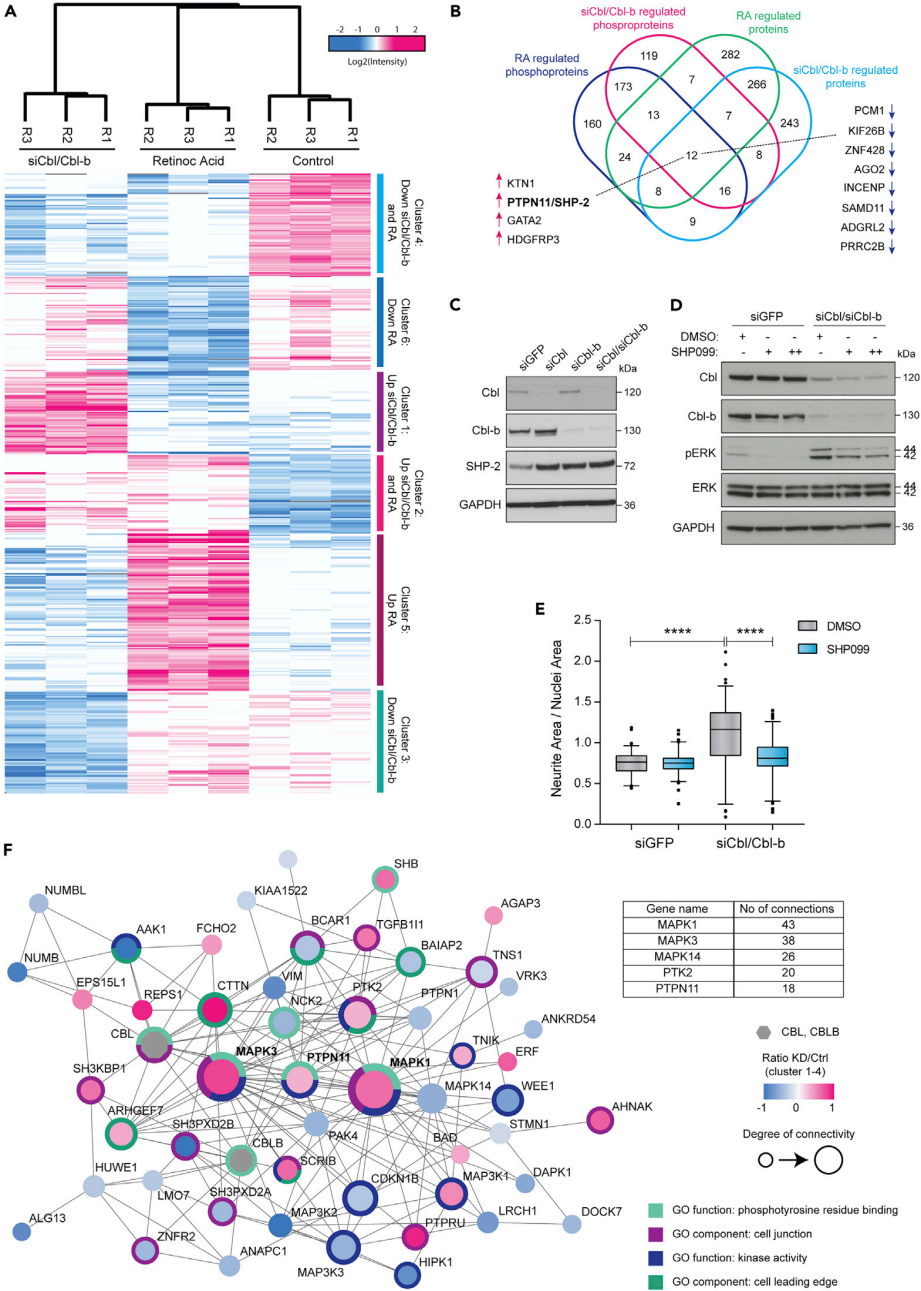
Phosphoproteomics identifies SHP-2 among the regulators of ERK-driven neurite outgrowth (A) Hierarchical clustering of phosphorylation sites differentially regulated (ANOVA) by treatment with Cbl/Cbl-b siRNA or retinoic acid (RA) for 24 hr. The six identified clusters selected for further analyses are highlighted. Data are presented with log_2_ normalized TMT intensities. (B) Venn diagram showing the overlap of phosphoproteins and proteins regulated by siCbl/Cbl-b and RA treatment. Common proteins are highlighted with arrows indicating upregulation/downregulation. (C) Immunoblotting of lysates from SH-SY5Y cells treated with siGFP, siCbl, siCbl-b, or both, detecting the indicated proteins. (D) Immunoblotting of lysates from SH-SY5Y cells treated with siGFP or siCbl/Cbl-b and SHP-2 inhibitor (SHP099; + and ++ indicates 5 or 10 μM, respectively) for 72 hr. (E) Quantification of neurite outgrowth based on the same conditions as in (D). Data are shown as medians with 95% CI. Data are representative of three independent experiments (n = 3). ∗∗∗∗ indicates p < 0.0001 (one-way ANOVA). (F) Cluster containing Cbl and Cbl-b (gray) detected by MCL clustering based on functional network analysis of proteins belonging to clusters 1-4. Node color indicates regulation at phosphosite level for knockdown (KD) relative to control. Most significantly overrepresented GO terms are indicated in outer circle and color legend. The table summarizes the number of connections for the most connected proteins.

As Cbl and Cbl-b are implicated in regulation of cellular signaling (Thien and Langdon, 2001), we reasoned that the absence of Cbl proteins might affect the long-term phosphorylation status of several other proteins involved in neurite outgrowth besides ERK. To globally assess changes in phosphorylation levels, we analyzed the phosphoproteome of Cbl/Cbl-b-depleted SH-SY5Y cells. We included an RA-treated condition to represent known differentiation signaling and help detect changes associated with neurite outgrowth. Samples were prepared in triplicates, and lysates were labeled by stable isotope-labeled tandem mass tags (TMTs) 10-plex and mixed prior to phosphopeptide enrichment with TiO2 beads and separation by offline high-pH reversed-phase fractionation before LC-MS/MS analysis (Figure S4A). We identified and quantified 15,480 phosphorylation sites, of which 12,640 were confidently localized (class I sites) to a specific serine (11,004), threonine (1544), or tyrosine (92) in the peptide sequence (Figures S4B and S4C and Table S3). The sites were distributed on 3439 phosphoproteins, and most peptides were identified as singly phosphorylated (Figures S4B and S4D). Furthermore, fractions of the samples were used for proteome profiling as described in Figure S4A. Hierarchical clustering separated both phosphoproteome and proteome data according to treatment (Figures S4E and S4F). ANOVA statistical testing identified 796 class I sites as being differentially phosphorylated between the three conditions. Hierarchical clustering separated these sites into six main clusters (Figure 4A and Table S3). Sites considered regulated by siCbl/Cbl-b treatment were found by combining clusters 1 and 2 (202 sites upregulated by Cbl/Cbl-b depletion) and clusters 3 and 4 (263 sites downregulated by Cbl/Cbl-b depletion). For RA treatment, we found 305 upregulated sites (clusters 2 and 5) and 253 downregulated sites (clusters 4 and 6). The same analysis was carried out for the proteome (Figure S4G and Table S4). To pursue the idea of potential common regulators of ERK activation and neurite outgrowth, we initially focused on the shared fraction of regulated phosphoproteins and proteins between Cbl/Cbl-b-depleted and RA-treated cells (Figure 4B). A total of 12 proteins were regulated both on the phosphoproteome and protein level of which 4 and 8 were upregulated and downregulated, respectively (see Figure 4B). We reasoned that central protein hubs in the Cbl-dependent network incorporate multilevel signals and are therefore regulated on several proteomic levels (Francavilla et al., 2016)*.* Within the upregulated group, we found the tyrosine phosphatase SHP-2 as an interesting candidate for further investigation. SHP-2 represents a known signaling hub for relaying signals from the cell surface to downstream effectors, has been connected to neurite outgrowth (Chen et al., 2002; Wright et al., 1997), and is a clinically relevant phosphatase considering that 14 phase 1 and phase 2 trials with SHP-2 allosteric inhibitor are currently running. By western blotting, we validated the increased protein levels of SHP-2 upon Cbl/Cbl-b knockdown observed in the proteome data (Figures 4C and 4B/S4G). SHP-2 has previously been linked to neurite outgrowth (Chen et al., 2002; Wright et al., 1997) and is known to be essential for full activation of the RAS/ERK pathway (Chan and Feng, 2007). Thus, we hypothesized that under normal conditions, Cbl proteins inhibit SHP-2 either directly or indirectly, thereby inhibiting ERK phosphorylation and neurite outgrowth. To test this hypothesis, we applied the recently developed first-in-class allosteric SHP-2 inhibitor SHP099 (Chen et al., 2016) to specifically target SHP-2 function in combination with Cbl and Cbl-b depletion and observed decreased levels of siCbl/Cbl-b-induced ERK phosphorylation upon SHP-2 inhibition (Figure 4D). This effect was associated with a relative decrease in the level of neurite outgrowth (Figure 4E). However, we still observed residual levels of ERK phosphorylation in response to Cbl/Cbl-b depletion in the presence of SHP099 (Figure 4D). Thus, we reasoned that the complete regulatory effect on ERK activation and neurite outgrowth mediated by Cbl proteins could not be ascribed to SHP-2-dependent signaling alone. Focusing our analysis on the total number of siCbl/Cbl-b-regulated phosphorylation sites (465), we performed a functional network analysis of phosphoproteins regulated by siCbl/Cbl-b treatment (clusters 1–4), which grouped these into two main clusters. One of these was highly connected to Cbl (and Cbl-b) and with functions in processes such as phosphotyrosine binding and kinase signaling. This cluster was centered on MAPK ERK1/2 as the most connected proteins and contained other proteins linked to MAPK signaling, including SHP-2/PTPN11 (Figure 4F). KEGG (Kyoto Encyclopedia of Genes and Genomes) pathway enrichment analysis of proteins with regulated phosphosites revealed an overrepresentation of proteins associated with e.g. signaling and cytoskeletal rearrangements (focal adhesion) for upregulated sites and cell cycle for downregulated sites (Figure S4H).

The partial effect of SHP-2 inhibition suggests that several interconnected signaling networks are needed to regulate ERK activity and neurite outgrowth and that the Cbl proteins seem to be central signaling hubs underlying this regulation.

### CDK16 is implicated in the regulation of ERK phosphorylation and neurite outgrowth in response to Cbl protein knockdown

**Figure 5:**
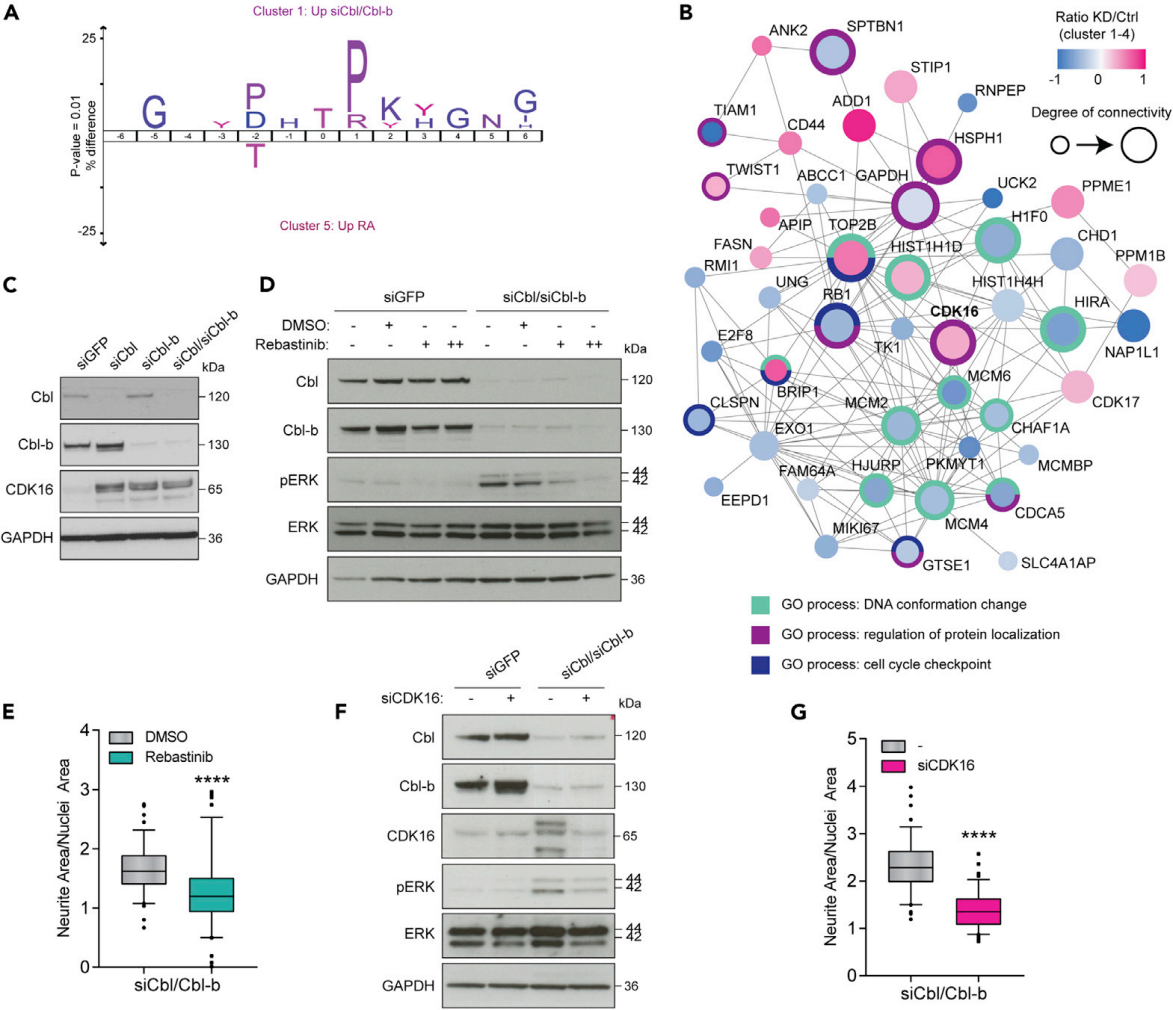
The inhibitory effect of Cbl/Cbl-b on neurite outgrowth depends on CDK16 activity (A) Sequence motif analysis (IceLogo) of the 6 amino acid residues flanking the regulated phosphorylation site as identified in Figure 4A, cluster 1 (KD-upregulated) compared to cluster 5 (RA-upregulated). (B) Second largest cluster detected by MCL clustering based on functional network analysis of proteins belonging to clusters 1-4 from Figure 4A. Node color indicates regulation at phosphosite level for KD relative to control. Most significantly overrepresented GO terms are indicated in outer circle and color legend. CDK16 is highlighted in bold. (C) Immunoblotting of lysates from SH-SY5Y cells treated with siGFP, siCbl, siCbl-b, or both, detecting the indicated proteins (same lysates as in Figure 4C). (D) Immunoblotting of lysates from SH-SY5Y cells treated with siGFP or siCbl/Cbl-b and CDK16 inhibitor (rebastinib; + and ++ indicates 0.5 or 1 μM, respectively) for 48–72 hr. Data are representative of three independent experiments (n = 3). (E) Representative quantification of neurite outgrowth based on the conditions in (D). (F) Immunoblotting of lysates from SH-SY5Y cells treated with siGFP or siCbl/Cbl-b and siCDK16 for 72 hr, using the indicated antibodies. (G) Quantification of neurite outgrowth based on the same conditions as in (F). Data in boxplots are shown as medians with 95% CI. Data are representative of two ([E] and [G]) or three (F) independent experiments (n = 2-3). ∗∗∗∗ indicates p < 0.0001 (t test).

To identify protein kinases implicated in the Cbl/Cbl-b-dependent effect on neurite outgrowth, we performed a sequence motif analysis (Colaert et al., 2009), which relies on the fact that the specificity of many kinases can be assigned to their preferred consensus sequence surrounding the phosphorylated residue. We focused on the upregulated sites as we expect increased activity of kinases functioning as drivers of this process. By comparing phosphorylation sites exclusively upregulated by Cbl/Cbl-b depletion (cluster 1; Figure 4A) to those exclusively upregulated by RA (cluster 5; Figure 4A), we found a strong preference of sites in cluster 1 for proline in the +1 position relative to the phosphorylated residue (Figure 5A). This observation points to a higher activation of proline-directed kinases in cells depleted of Cbl and Cbl-b. The group of well-established proline-directed kinases includes kinase families related to MAPK signaling (such as ERK and p38), glycogen synthase kinase-3 (GSK3), and cyclin-dependent kinases (CDKs).

Among the kinases upregulated in response to Cbl/Cbl-b depletion, we identified the CDK CDK16, as part of the second largest cluster in the functional network analysis (Figure 5B). This cluster contained proteins mostly related to DNA and cell cycle regulation. We found phosphorylation of CDK16 on a regulatory site Ser119 to be exclusively upregulated by Cbl/Cbl-b knockdown (cluster 1 in Figure 4A). Furthermore, the protein levels of CDK16 were also significantly increased by knockdown of Cbl proteins in the 72 hr proteome data set (cluster A in Figure 2D and Table S1). The increase in CDK16 protein levels upon Cbl protein knockdown was subsequently validated by western blotting (Figure 5C). CDK16 is not very well studied but has been linked to different signaling pathways and processes such as exocytosis, spermatogenesis, autophagy, and neuronal development (Dohmen et al., 2020; Malumbres, 2014; Mokalled et al., 2010). Thus, we considered that this kinase might mediate the siCbl/Cbl-b-dependent effects on ERK and neurite outgrowth. To further investigate the functional role of CDK16 and establish a link to Cbl protein function, we used the pharmacological inhibitor rebastinib, which has been shown to target CDK16 (Dixon-Clarke et al., 2017). We treated the neuroblastoma cells in combination with siCbl/Cbl-b treatment and observed that the effect of Cbl/Cbl-b depletion on ERK phosphorylation was abolished by rebastinib treatment (Figure 5D). This also correlated with an observed decrease in the levels of neurite outgrowth (Figure 5E). Rebastinib is currently in three phase 1 or 2 clinical trials, as combinatorial treatment, and is commonly used to target the tyrosine kinase Abl1. To check for potential effects of other targets, we investigated the protein expression of Abl1 as well as other potential rebastinib off-targets as reported by the supplier. Four of these proteins were not found to be expressed in the SH-SY5Y cells by our deep proteome analysis, while the other five were detected, yet none of these were specifically regulated by Cbl/Cbl-b depletion (Figure 2D; cluster A and B). Src and Lyn, however, were found in the commonly upregulated and downregulated clusters, respectively, displaying large ratios for cocktail ligand stimulation but only very small ratios for Cbl/Cbl-b depletion (Figure S5A). We identified several phosphosites on some of these potential off-targets; however, not a single site on any of the proteins was regulated by Cbl/Cbl-b knockdown (Figure S5A). Overall, we concluded that reported rebastinib off-targets were not responsible for the Cbl protein-dependent effects on ERK regulation and neurite outgrowth in our system. To confirm that rebastinib targeted CDK16 in the neuroblastoma cells, we treated SH-SY5Y cells with rebastinib in a concentration based on the determined IC50 value (Figure S5B) and performed a cellular thermal shift assay (Jafari et al., 2014). Immunoblotting of rebastinib or dimethyl sulfoxide (DMSO)-treated cells for CDK16 revealed increased stability of CDK16 in lysates of cells treated with the inhibitor compared to vehicle (Figure S5C). This indicated that rebastinib binds to CDK16 in SH-SY5Y. To further validate a functional role of CDK16 in ERK phosphorylation and neurite outgrowth, we treated Cbl/Cbl-b-depleted cells with siRNAs targeting CDK16. We observed that treatment with CDK16 siRNAs blocked the siCbl/Cbl-b-induced increase in CDK16 protein and that this correlated with decreased levels of ERK phosphorylation and neurite outgrowth (Figures 5F and 5G). These data align with the results of the rebastinib treatment and support the theory of CDK16 playing a role in the Cbl-mediated effects on ERK phosphorylation and neurite outgrowth in neuroblastoma cells. To examine whether the regulatory role of Cbl and Cbl-b observed in SH-SY5Y could be relevant to neuroblastoma in general, we depleted Cbl and Cbl-b in two other neuroblastoma cell lines, Kelly and NBL-S, to assess the effects on protein levels of CDK16, SHP-2, and IGF1R as well as the effects on ERK phosphorylation and neurite outgrowth. Knockdown of Cbl/Cbl-b in both cell lines resulted in increased levels of CDK16, SHP-2, and IGF1R in line with the data from SH-SY5Y (Figure S5D). This was accompanied by increased phospho-ERK and a trend toward increased neurite outgrowth, although the morphological changes in the phenotype were not as evident as for SH-SY5Y (Figures S5D and S5E). These data suggest a general link between Cbl E3 ligases, CDK16 and SHP-2, and ERK phosphorylation in neuroblastoma.

## Discussion

**Figure 6:**
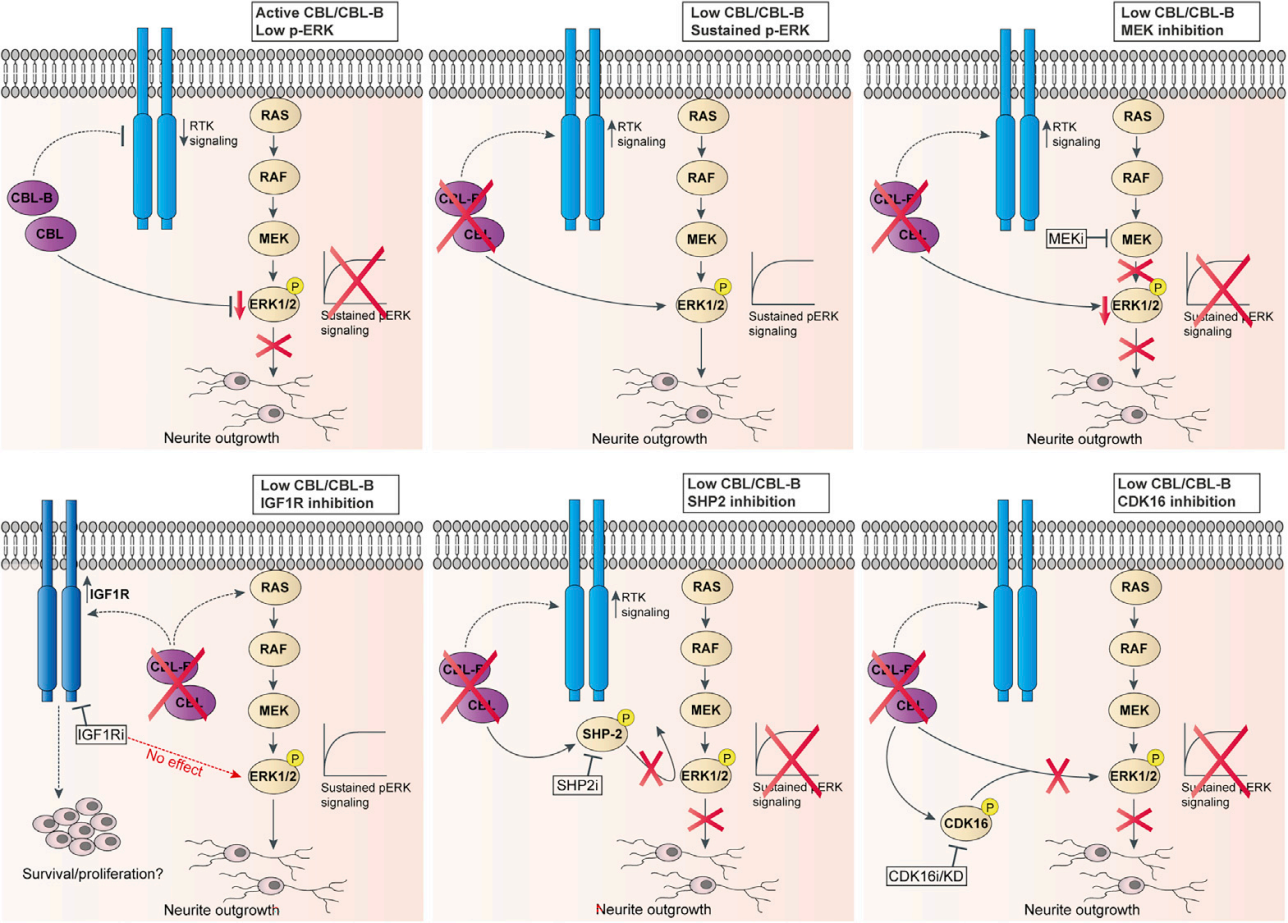
Schematic of the role of Cbl and Cbl-b and associated signaling proteins in regulation of ERK phosphorylation and neurite outgrowth in neuroblastoma cells Models summarizing the role of Cbl and Cbl-b in negative regulation of ERK phosphorylation and neurite outgrowth in the SH-SY5Y neuroblastoma cells. The schematics illustrate the identified signaling proteins and how inhibition of these proteins revealed their implication in Cbl/Cbl-b-dependent regulation of ERK phosphorylation and neurite outgrowth.

In this study, we investigated the role of Cbl proteins (Cbl and Cbl-b) in regulation of neuroblastoma cell differentiation by using large-scale proteomics analysis of long-term signaling responses in combination with biochemical and functional assays. Through this approach, we identified proteins such as the tyrosine phosphatase SHP-2 and the CDK CDK16 to be involved in Cbl protein-mediated regulation of sustained ERK phosphorylation and neurite outgrowth in SH-SY5Y cells (Figure 6).

This investigation was prompted by our previous discovery that individual Cbl proteins inhibit neurite outgrowth in neuroblastoma cells (Emdal et al., 2015). Initially, we found an increased effect on neurite outgrowth and ERK phosphorylation in neuroblastoma cell lines by simultaneous depletion of Cbl and Cbl-b compared to individual knockdown. An additive effect of depleting both Cbl and Cbl-b has also been shown for Cbl protein-mediated regulation of ubiquitylation and activation of RTKs, exemplified by PDGFRβ (Rorsman et al., 2016).

The role of Cbl proteins in activation-induced regulation of RTK stability and signaling is well established (Ettenberg et al., 2001; Miyake et al., 1998; Petrelli et al., 2002; Wong et al., 2002; Yokouchi et al., 1999); thus, our initial hypothesis focused on the involvement of RTKs in Cbl protein-dependent regulation of sustained ERK phosphorylation and neurite outgrowth. We were intrigued to find that basal IGF1R protein levels were increased upon Cbl depletion; however, inhibition of IGF1R activity did not abolish the increase in ERK phosphorylation and neurite outgrowth (Figure 6). This was observed despite an increase in neurite outgrowth upon IGF1R ligand-induced activation. Cbl proteins have previously been found to regulate IGF1R ubiquitylation and turnover in response to ligand stimulation (Li et al., 2014; Sehat et al., 2008). The increased IGF1R levels upon Cbl knockdown could potentially be coupled with increased neuroblastoma cell viability or proliferation, as we do observe a slightly higher dependency on IGF1R for cell survival of Cbl/Cbl-b-depleted SH-SY5Y cells treated with IGF1R inhibitor (Figure S3G). Indeed, IGF1R inhibition has been associated with decreased neuroblastoma cell growth and shown potent anti-tumor effects (Guerreiro et al., 2006; Tanno et al., 2006; Van den Eynden et al., 2018; Zhao et al., 2015). Thus, there could be a rationale for further investigations into the possibilities of combining IGF1R inhibition with differentiation therapy through modulation of Cbl protein signaling networks for the treatment of neuroblastoma.

Through our analysis of global long-term phospho-signaling, we identified the tyrosine phosphatase SHP-2 as upregulated both on phosphosite and protein level in response to Cbl protein depletion and RA treatment. This pointed to a general role of SHP-2 in neuroblastoma cell differentiation. Indeed, this oncogenic phosphatase is implicated in the regulation of RTK signaling responses and neurite outgrowth (Chen et al., 2002; Hanafusa et al., 2004; Perrinjaquet and Iba, 2010; Wright et al., 1997). While inhibition of SHP-2 with the inhibitor SHP099 (Chen et al., 2016) partially reversed the effect of Cbl protein depletion on ERK phosphorylation and neurite outgrowth (Figure 6), the full effects could not be explained by SHP-2 regulation alone. Our phosphoproteome data set also pointed to a potential role of CDK16 in Cbl protein-dependent regulation of neurite outgrowth supported by the 72-hr deep proteome data in which CDK16 was identified as one of the top three most upregulated kinases. We found that inhibition of CDK16 with rebastinib or siRNA-mediated knockdown of CDK16 abolished ERK phosphorylation and neurite outgrowth in response to Cbl protein depletion. CDK16 belongs to the PCTAIRE subfamily of CDKs. CDK16 has previously been connected to neurite outgrowth regulation; thus, siRNA-based knockdown of CDK16 has been shown to disrupt dendrite development as well as neurite outgrowth in primary neuron cultures (Fu et al., 2011; Graeser et al., 2002; Mokalled et al., 2010). Intriguingly, the PCTAIRE family has recently been highlighted as a significantly understudied CDK group of oncology drug targets (Axtman et al., 2019). Here, we report an association of CDK16 to ERK and Cbl protein-regulated signaling pathways, linking endogenous CDK16 to ERK phosphorylation and neurite outgrowth in neuroblastoma cells (Figure 6). Interestingly, we also observed increased phosphorylation of the closely related and even less studied family member CDK17 (Figure 5B and Table S3); thus, it could be speculated that this kinase exerts similar functions.

A conundrum of cell signaling is the concept of functional selectivity; how receptor-mediated activation of the same signaling pathways can lead to distinct cellular outcomes, as previously addressed with MS-based proteomics (Francavilla et al., 2013, 2016). Extensive studies in PC12 cells have shown that nerve growth factor (NGF) stimulation induces neurite outgrowth and differentiation, while epidermal growth factor (EGF) stimulation is associated with a strictly mitogenic response (Marshall, 1995; Traverse et al., 1992). These effects have been attributed to the differential NGF- and EGF-dependent signaling dynamics inducing sustained and transient ERK phosphorylation, respectively. The differential ERK signaling regulation and responses in PC12 cells have been explained by NGF mediating positive feedback loops from MAPK to Raf, whereas EGF only mediated negative feedback (Grewal et al., 1999; Kao et al., 2001; Santos et al., 2007). It has been shown that changes in phospho-ERK signaling at 24 hr of stimulation, but not at 5 min, had a predictive value for the neuronal differentiation and proliferation state at 48 hr, implying that sustained, but not short-term signals, ultimately determine cell fate outcomes (Chen et al., 2012). This illustrates the complex nature of ERK signaling and the importance of fine-tuned regulation for determination of cellular fate. Elucidating the exact molecular mechanisms mediating the sustained ERK phosphorylation response induced by Cbl protein depletion could likely depend on the development of other ways to manipulate Cbl function, in particular development of small-molecule inhibitors to target Cbl/Cbl-b. Our multi-level proteomics approach provides insights into how Cbl proteins regulate long-term global signaling responses related to sustained ERK phosphorylation and neurite outgrowth. We identify different proteins for which we functionally validate the role in the neurite outgrowth response induced by Cbl protein depletion. Thus, we hope our data can help to increase the understanding of the complex process of neuroblastoma cell differentiation and provide new avenues for further in-depth investigation.

### Limitations of the study

The high level of heterogeneity characterizing neuroblastoma constitutes a challenge when studying this disease. Accordingly, observations such as those made in the present study might relate to subtypes of neuroblastoma tumors. Thus, while we do observe similar effects of Cbl and Cbl-b depletion in a panel of neuroblastoma cell lines, the magnitude of the effects differ between cell lines. This could potentially be due to intrinsic differences in protein expression and activity levels in the neuroblastoma cells and/or differential expression of known neuroblastoma oncogenes such as MYCN or ALK. For instance, ALK is amplified in the NB1 cells and known to drive their growth, which could explain the relatively high background level of ERK phosphorylation and the less obvious effect of Cbl/Cbl-b knockdown in these cells. However, this warrants further study. Differences in protein expression levels between cell lines can potentially also relate to different levels of dependency on Cbl proteins, which would result in varying effects of their depletion. Naturally, there is also an intrinsic limitation when using a model system based on siRNA-mediated depletion of target proteins as it is not possible to obtain complete protein ablation and there might be differences in transfection efficiency between cell lines. However, based on the need of this study to be performed with transient and simultaneous knockdown of Cbl and Cbl-b to induce a differentiation response, siRNA-based depletion was found to be the best approach. The need for approximately 24 h of siRNA treatment to yield sufficient knockdown levels constitutes another limitation in this type of study as compared to e.g. inhibitor-based studies. Accordingly, development of small-molecule inhibitors targeting Cbl/Cbl-b would enable characterization of short-term responses including changes in protein interaction partners and more immediate changes in global ubiquitylation and phosphorylation, which could aid interpretation of the long-term responses observed in the current manuscript.

### Resource availability

#### Lead contact

Further information and requests for resources should be directed to and will be fulfilled by the lead contact, Dr. Jesper V. Olsen (jesper.olsen@cpr.ku.dk).

#### Materials availability

This study did not generate any new unique reagents.

#### Data and code availability

The accesion number for the mass spectrometry proteomics data reported in this paper is [ProteomeXchange Consortium via PRIDE (Perez-Riverol et al., 2019)]: [PXD020389].

## Methods

All methods can be found in the accompanying transparent methods supplemental file.

## Supplemental information

Document S1. Transparent methods and Figures S1–S5

Table S1. SILAC 72 hr proteome list of identified and regulated proteins, related to Figures 2, S2, and S3

Table S2. SILAC 24 hr ubiquitylome list of identified and regulated ubiquitin sites, related to Figures 2 and S3

Table S3. TMT 24 hr phosphoproteome list of identified and regulated phosphorylation sites, related to Figures 4, 5, and S4

Table S4. TMT 24 hr proteome list of identified and regulated proteins, related to Figures 4 and S4
