# Supplementary material for: Proteomic investigation of Cbl and Cbl-b in neuroblastoma cell differentiation highlights roles for SHP-2 and CDK16
Source: iScience. 2021 Mar 17;24(4):102321. doi: 10.1016/j.isci.2021.102321 (PMC8050387; doi:10.1016/j.isci.2021.102321)
Supplement: Document S1. Transparent methods and Figures S1–S5 [file mmc1.pdf]

iScience, Volume ■■

Supplemental information

Proteomic investigation of Cbl and Cbl-b in neuroblastoma cell differentiation highlights roles for SHP2 and CDK16

Anna-Kathrine Pedersen, Anamarija Pfeiffer, Gopal Karemore, Vyacheslav Akimov, Dorte B. Bekker-Jensen, Blagoy Blagoev, Chiara Francavilla, and Jesper V. Olsen

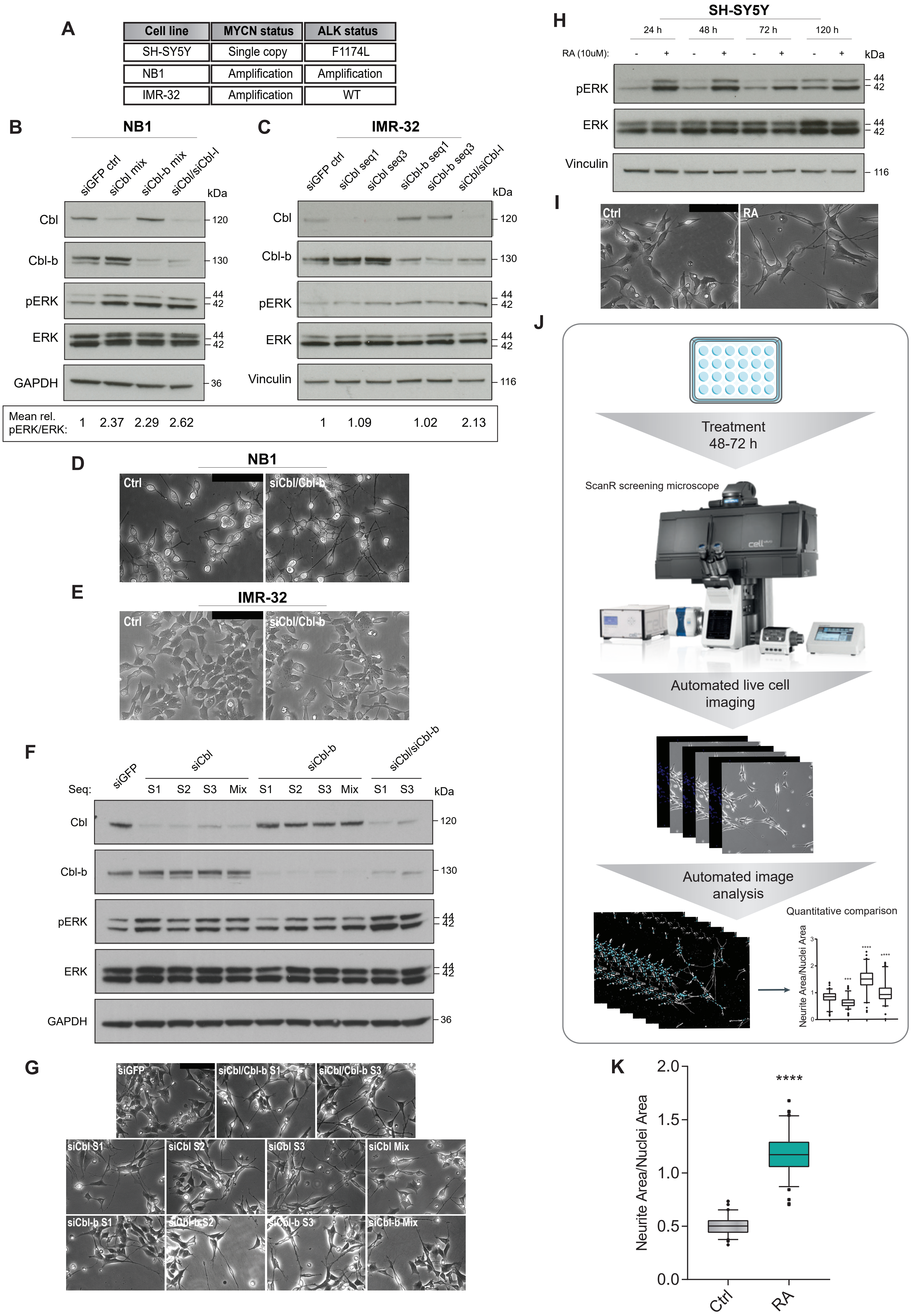

A
Cell line
MYCN status
ALK status
SH-SY5Y
Single copy
F1174L
NB1
Amplification
Amplification
IMR-32
Amplification
WT
B
NB1
siGFP ctrl
siCbl mix
siCbl-b mix
siCbl/siCbl-l
kDa
Cbl
Cbl-b
pERK
ERK
GAPDH
120
130
44
42
36
Mean rel. pERK/ERK:
1
2.37
2.29
2.62
C
IMR-32
siGFP ctrl
siCbl seq1
siCbl seq3
siCbl-b seq1
siCbl-b seq3
siCbl/siCbl-l
Vinculin
116
1
1.09
1.02
2.13
D
NB1
Ctrl
siCbl/Cbl-b
E
IMR-32
F
siGFP
siCbl
siCbl-b
siCbl/siCbl-b
Seq:
S1
S2
S3
Mix
G
siGFP
siCbl/Cbl-b S1
siCbl/Cbl-b S3
siCbl S1
siCbl S2
siCbl S3
siCbl Mix
siCbl-b S1
siCbl-b S2
siCbl-b S3
siCbl-b Mix
H
SH-SY5Y
24 h
48 h
72 h
120 h
RA (10uM):
I
Ctrl
RA
J
Treatment
48-72 h
ScanR screening microscope
Automated live cell imaging
Automated image analysis
Quantitative comparison
Neurite Area/Nuclei Area
K
2.0
1.5
1.0
0.5
0.0
****
Neurite Area/Nuclei Area
Ctrl
RA

Figure S1. Depletion of Cbl/Cbl-b and RA stimulation leads to increased ERK phosphorylation levels associated with increased neurite outgrowth in neuroblastoma cell lines, Related to Figure 1. **(A)** Table of the three applied neuroblastoma cell lines. **(B-C)** Lysates from NB1 **(B)** or IMR-32 **(C)** cells treated with control siRNA or siRNA against Cbl and/or Cbl-b for 48-72 hours, were immunoblotted detecting the indicated proteins and quantified as shown below. Data are representative of two independent experiments (n=2). **(D-E)** Representative images showing visualization of neurite outgrowth of NB1 and IMR32 cells, respectively, treated with Ctrl or Cbl/Cbl-b siRNA, (scale bar, 50 µm). **(F-G)** SH-SY5Y cells were treated with siRNA against Cbl and/or Cbl-b (as indicated) for 72 hours. **(F)** Lysates were subjected to immunoblotting with antibodies against phospho-ERK/ERK or Cbl/Cbl-b. **(G)** Representative images for neurite outgrowth visualization (same conditions as in F). Data are representative of three independent experiments (n=3) **(H)** Lysates from SH-SY5Y cells treated with 10 µM RA for 24 to 120 hours, were immunoblotted detecting the indicated proteins. **(I)** Representative images for visualization of neurite outgrowth of SH-SY5Y cells after 72 hours of RA treatment. **(J)** Workflow illustrating automated neurite outgrowth analysis. **(K)** Quantification of neurite outgrowth of SH-SY5Y cells after 72 h of treatment with RA or DMSO. Data are shown as medians w. 95% CI and representative of two independent experiments (n=2). **** indicates P<0.0001 (t-test).

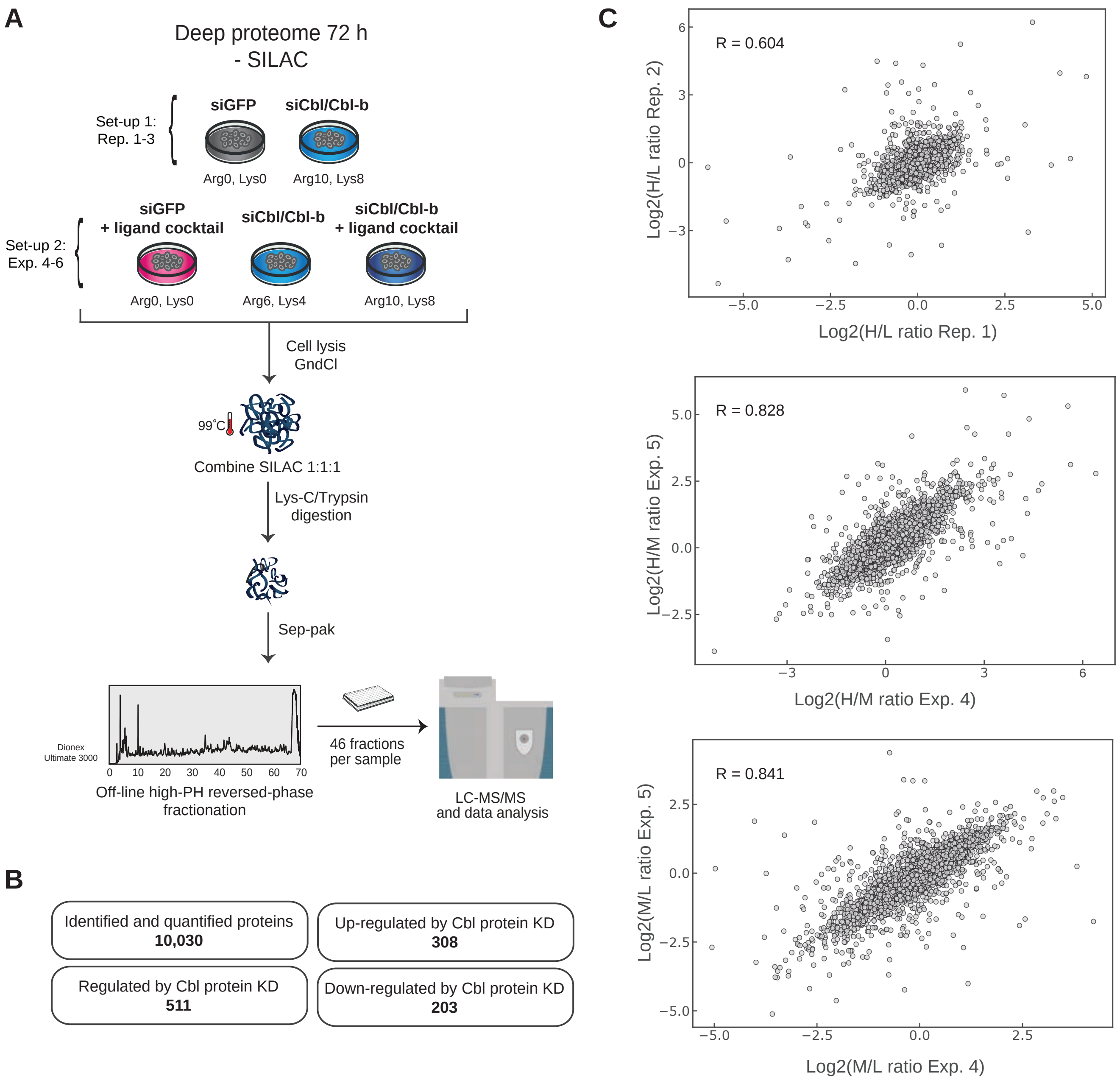

Figure S2. Deep proteome analysis of SH-SY5Y cells shows good quality controls, Related to Figure 2 and Table S1. (A) Experimental design and workflow of SILAC-based deep proteome analysis of Cbl and Cbl-b dependent long-term signaling in SH-SY5Y cells. Cells were treated with control or Cbl/Cbl-b siRNA combined with stimulation with RTK ligand cocktail (FGF-2, IGF-1, PDGF-BB, TGFα) for a total of 72 hours as indicated. **(B)** Overview of proteome data showing the numbers of identified proteins and proteins regulated by Cbl protein knockdown (as presented in Fig. 2D). **(C)** Representative correlation plots of normalized SILAC protein ratios between biological replicates with R indicating Pearson correlation coefficients.

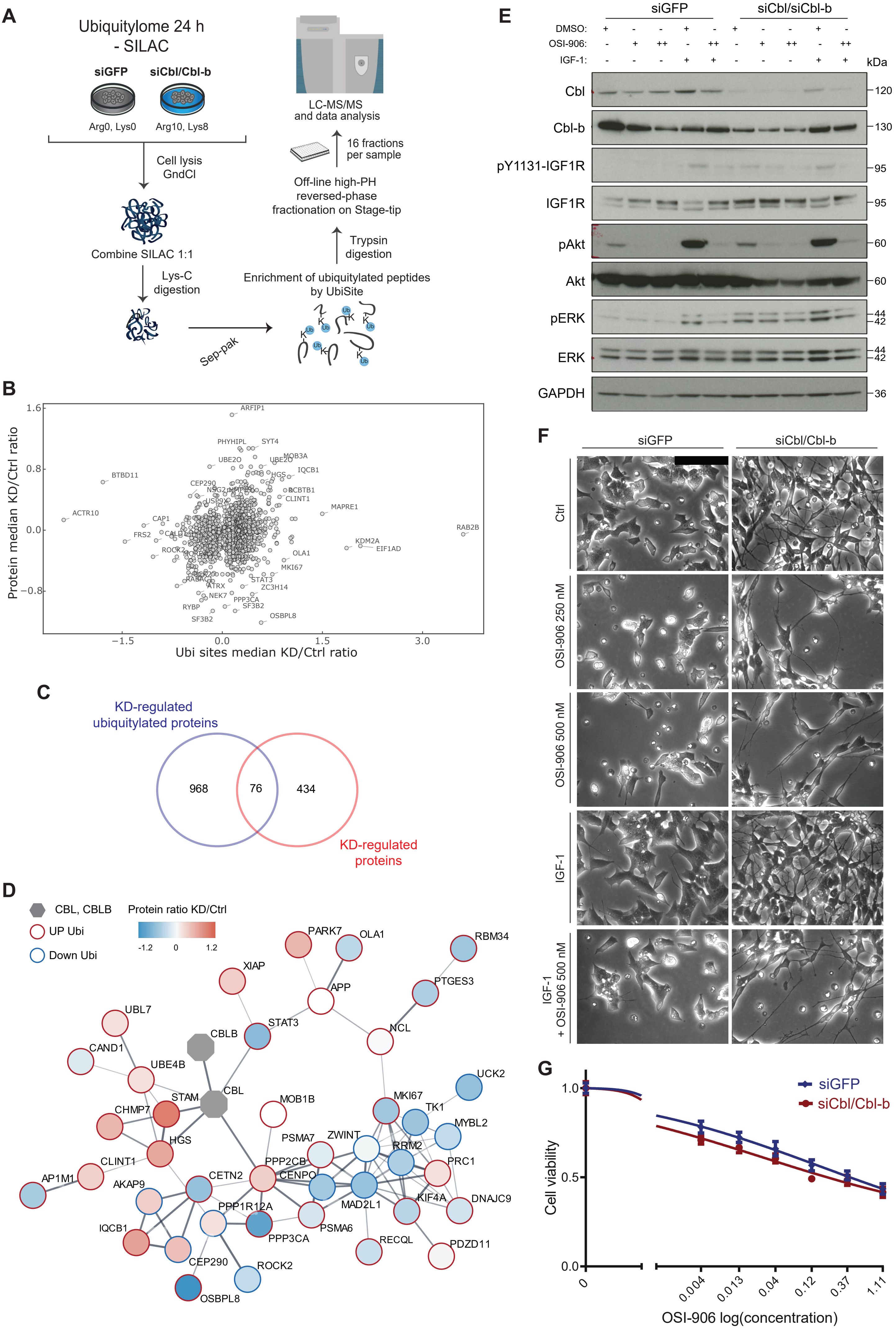
A
Ubiquitylome 24 h - SILAC
siGFP
siCbl/siCbl-b
Arg0, Lys0
Arg10, Lys8
Cell lysis GndCl
Combine SILAC 1:1
Lys-C digestion
Sep-pak
Enrichment of ubiquitylated peptides by UbiSite
Trypsin digestion
Off-line high-PH reversed-phase fractionation on Stage-tip
16 fractions per sample
LC-MS/MS and data analysis
B
Protein median KD/Ctrl ratio
Ubi sites median KD/Ctrl ratio
ARFIP1
PHYHIPL
SYT4
UBE2O
MOB3A
IQCB1
BTBD11
CEP290
HGS
KBTB1
CLINT1
MAPRE1
ACTR10
CAP1
FRS2
RAB2B
KDM2A
EIF1AD
OLA1
MKI67
STAT3
ZC3H14
ATRX
NEK7
PPP3CA
SF3B2
RYBP
OSBPL8
C
KD-regulated ubiquitylated proteins
968
76
434
KD-regulated proteins
D
CBL, CBLB
UP Ubi
Down Ubi
Protein ratio KD/Ctrl
-1.2
0
1.2
PARK7
OLA1
RBM34
XIAP
APP
PTGES3
UBL7
STAT3
CBLB
NCL
CAND1
UBE4B
UCK2
STAM
CBL
MKI67
MOB1B
TK1
CHMP7
MYBL2
HGS
PSMA7
ZWINT
RRM2
CLINT1
PPP2CB
PRC1
AP1M1
CETN2
CENPO
AKAP9
KIF4A
DNAJC9
PPP1R12A
MAD2L1
IQCB1
PSMA6
PPP3CA
RECQL
PDZD11
CEP290
ROCK2
OSBPL8
E
siGFP
siCbl/siCbl-b
DMSO:
OSI-906:
IGF-1:
kDa
Cbl
Cbl-b
pY1131-IGF1R
IGF1R
pAkt
Akt
pERK
ERK
GAPDH
120
130
95
95
60
60
44
42
44
42
36
F
siGFP
siCbl/Cbl-b
Ctrl
OSI-906 250 nM
OSI-906 500 nM
IGF-1
IGF-1 + OSI-906 500 nM
G
siGFP
siCbl/Cbl-b
Cell viability
OSI-906 log(concentration)
1.0
0.5
0.0
0
0.004
0.013
0.04
0.12
0.37
1.11

Figure S3. The ubiquitylome of Cbl/Cbl-b-depleted SH-SY5Y cells and the role of IGF1R activity in Cbl/Cbl-b-mediated control of ERK phosphorylation and neurite outgrowth, Related to Figure 3 and Table S1 and S2. (A) Experimental workflow for ubiquitylome analysis of SH-SY5Y cells depleted of Cbl and Cbl-b. **(B)** Correlation between ratios of regulated ubiquitin sites and the ratio of their corresponding protein as identified in the 72 hour-proteome data. Selected gene names are indicated. **(C)** Venn diagram showing the overlap between proteins with regulated ubiquitylation sites and proteins regulated at 72 hours by siCbl/Cbl-b treatment. **(D)** Cbl/Cbl-b connected network as identified by functional network analysis of the 76 shared regulated proteins found in (C). Node color indicates regulation at protein level and outer circle indicates regulation of ubiquitin sites (blue: Down, red: Up). **(E)** Immunoblotting of lysates from SH-SY5Y cells treated with siGFP or siCbl/Cbl-b and IGF1R inhibitor (Linsitinib/OSI-906; + and ++ indicates 250 or 500 nM, respectively) for 72 hours, using the indicated antibodies. **(F)** Representative images based on the same conditions as in (E), (scale bar, 50 µm). Data are representative of three independent experiments (n=3). **(G)** Cell viability of SH-SY5Y siCbl/Cbl-b or siGFP-treated cells in response to increasing doses of OSI-906 for 72 h. Data is presented as means ± SEM, (n=3).

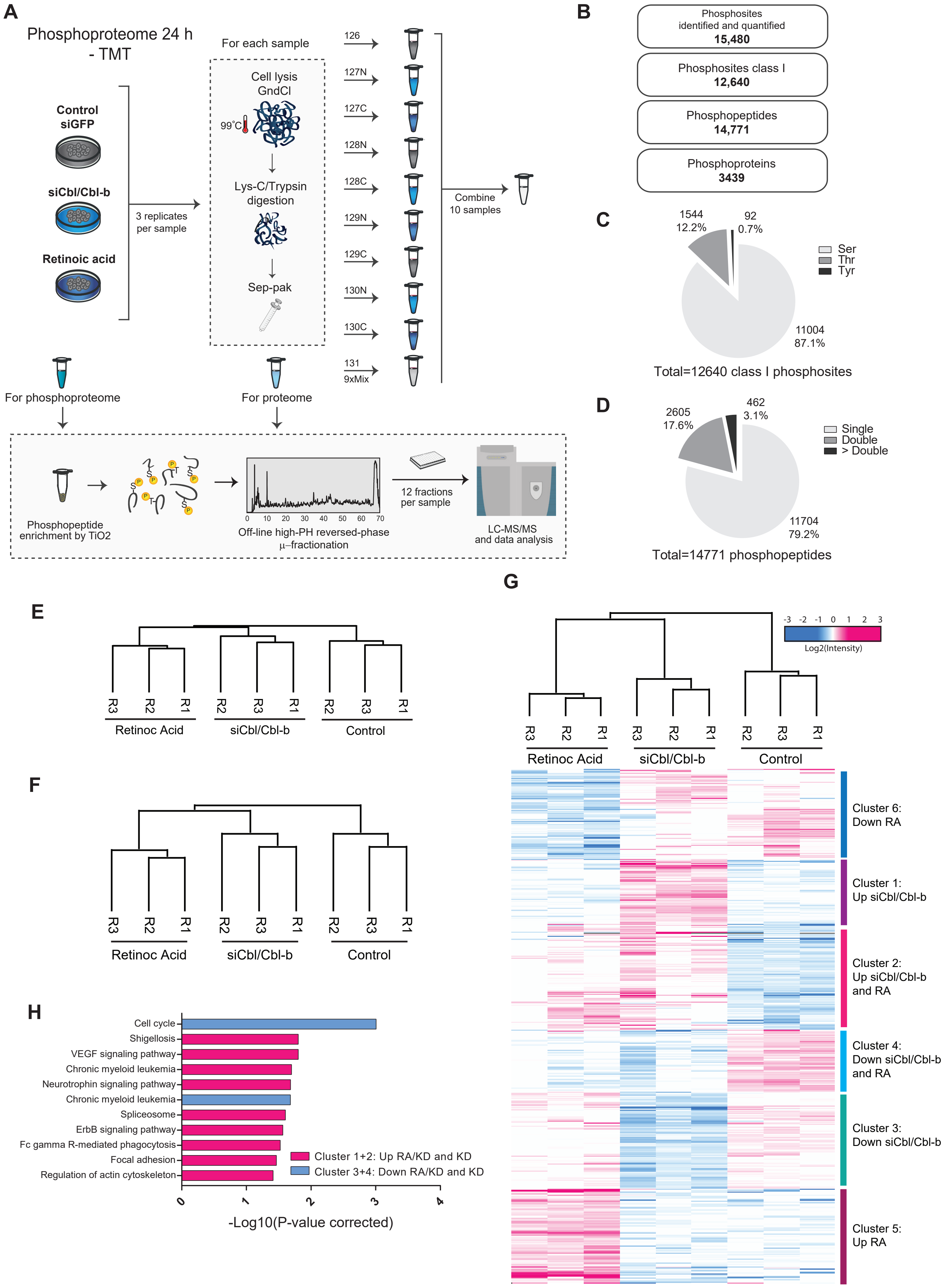
A
Phosphoproteome 24 h - TMT
Control siGFP
siCbl/Cbl-b
Retinoic acid
3 replicates per sample
For each sample
Cell lysis GndCl
99˚C
Lys-C/Trypsin digestion
Sep-pak
126
127N
127C
128N
128C
129N
129C
130N
130C
131 9xMix
Combine 10 samples
For phosphoproteome
For proteome
Phosphopeptide enrichment by TiO2
Off-line high-PH reversed-phase µ–fractionation
12 fractions per sample
LC-MS/MS and data analysis
B
Phosphosites identified and quantified 15,480
Phosphosites class I 12,640
Phosphopeptides 14,771
Phosphoproteins 3439
C
1544 12.2%
92 0.7%
11004 87.1%
Ser
Thr
Tyr
Total=12640 class I phosphosites
D
2605 17.6%
462 3.1%
11704 79.2%
Single
Double
> Double
Total=14771 phosphopeptides
E
Retinoc Acid
siCbl/Cbl-b
Control
F
Retinoc Acid
siCbl/Cbl-b
Control
G
Log2(Intensity)
Retinoc Acid
siCbl/Cbl-b
Control
Cluster 6: Down RA
Cluster 1: Up siCbl/Cbl-b
Cluster 2: Up siCbl/Cbl-b and RA
Cluster 4: Down siCbl/Cbl-b and RA
Cluster 3: Down siCbl/Cbl-b
Cluster 5: Up RA
H
Cell cycle
Shigellosis
VEGF signaling pathway
Chronic myeloid leukemia
Neurotrophin signaling pathway
Chronic myeloid leukemia
Spliceosome
ErbB signaling pathway
Fc gamma R-mediated phagocytosis
Focal adhesion
Regulation of actin cytoskeleton
Cluster 1+2: Up RA/KD and KD
Cluster 3+4: Down RA/KD and KD
-Log10(P-value corrected)

Figure S4. Overview of 24-hour phosphoproteome and proteome analysis of Cbl/Cbl-b depleted and RA stimulated SH-SY5Y cells, Related to Figure 4 and Table S3 and S4. (A) Experimental workflow for TMT10plex-based phosphoproteome and proteome analysis of SH-SY5Y cells depleted for Cbl and Cbl-b or treated with RA for 24 hours. **(B)** Overview of phosphoproteomics data. **(C)** Distribution of identified class I phosphorylation sites by amino acid. **(D)** Distribution of identified phosphopeptides with one, two or more than two phosphorylated sites. **(E-F)** Quality control of phosphoproteome data **(E)** and proteome data **(F)** by unsupervised hierarchical clustering using normalized intensities for identified and quantified phosphorylation sites or proteins, respectively. **(G)** Hierarchical clustering of proteins with significantly regulated abundance (ANOVA) in response to treatment with Cbl/Cbl-b siRNA or RA for 24 hours. The six identified clusters selected for further analyses are highlighted on the right. Data are presented with log2 normalized TMT intensities. **(H)** Bar graph showing results of KEGG pathway enrichment analysis for phospho clusters 1+2 (pink) and 3+4 (blue).

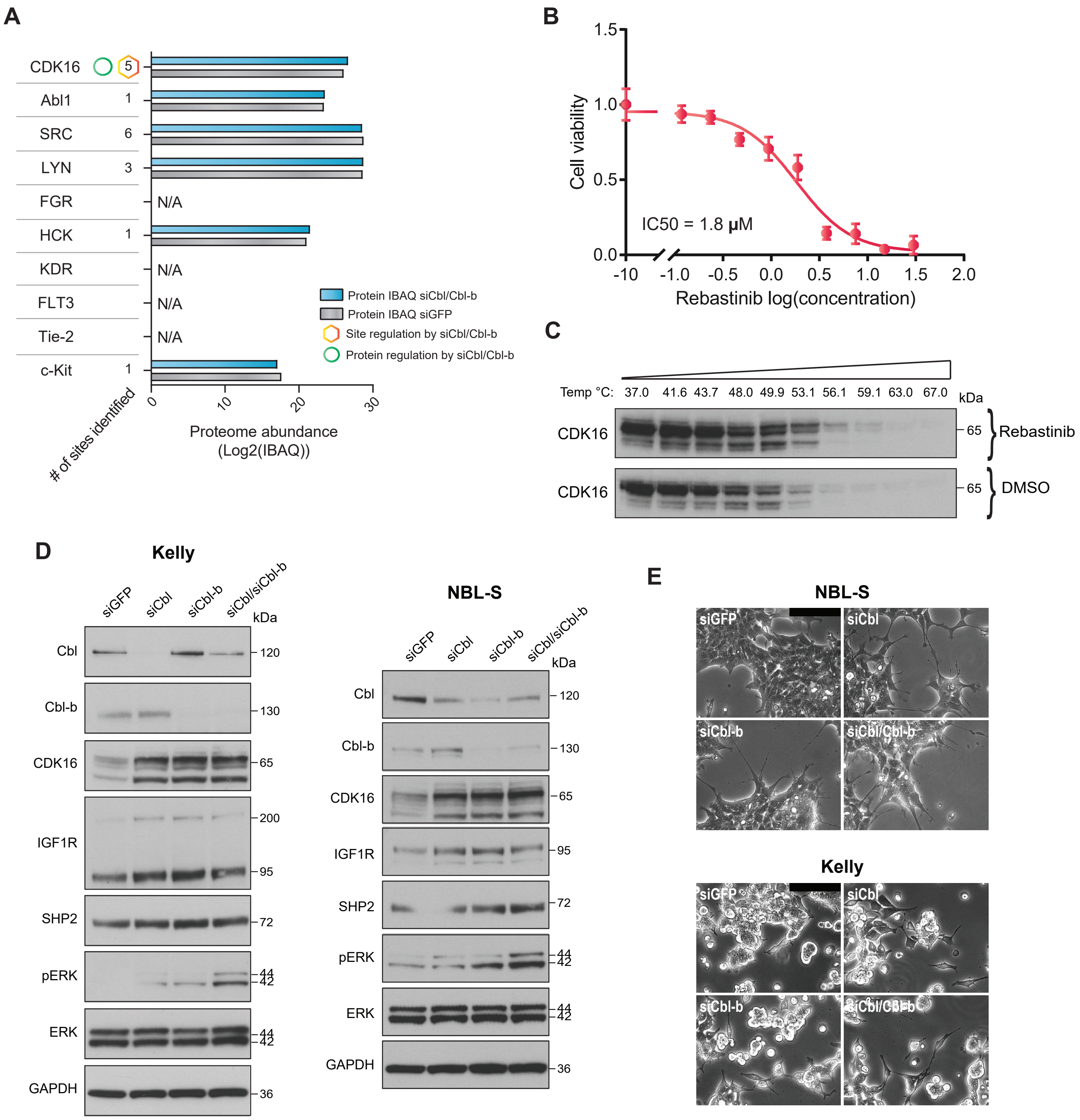

Figure S5. Rebastinib targets CDK16 in SH-SY5Y cells, Related to Figure 5. (A) Bar graph showing protein abundance (Log2(IBAQ) values) of Rebastinib off-targets and CDK16. N/A: Not available. The number of identified phosphorylation sites for each protein is indicated and circles indicate if a site or protein was identified as regulated by Cbl/Cbl-b knockdown. **(B)** Cell viability of SH-SY5Y cells in response to treatment with different concentrations of Rebastinib for 48 h for IC50 determination (IC50=1.8 μM). Data is presented as means ± SEM of 4 replicates. **(C)** For cellular thermal shift assay aliquots of Rebastinib- or DMSO-treated cells were heated at the indicated temperatures and subjected to immunoblotting with antibody against CDK16. **(D-E)** Kelly and NBL-S cells were treated with siRNA against Cbl and/or Cbl-b (as indicated) for 72 hours. **(D)** Lysates were subjected to immunoblotting with antibodies against the indicated proteins. **(E)** Representative images for neurite outgrowth visualization (same conditions as in D), (scale bar, 50 μm). Data is representative of three independent experiments (n=3).

Transparent Methods

Cell Culture and SILAC Labeling

The human neuroblastoma cell lines SH-SY5Y, NB1, NBL-S, Kelly and IMR-32 were cultured in RPMI 1640 with 2 mM L-glutamine (Gibco) supplemented with 10% fetal bovine serum (Gibco) and penicillin-streptomycin (100 U/mL and 100 µg/ml, Gibco). Cells were maintained at 37°C in a humidified incubator with 5% CO_2. All cell lines were tested negative for mycoplasma. For stable isotope labeling by amino acids in cell culture (SILAC)-based quantitative mass spectrometry, SH-SY5Y cells were grown and labeled for a minimum of two weeks in SILAC RPMI (PAA Laboratories), to yield three differentially labeled cell populations: Light Arg0/Lys0 (labeled with natural variants of Arginine and Lysine), medium Arg6/Lys4 (labeled with medium variants of the amino acids L-[13C6]Arg (+6) and L-[2H4]Lys (+4) and heavy Arg10/Lys8 (labeled with heavy variants of the amino acids L-[13C6,15N4]Arg (+10) and L-[13C6,15N2]Lys (+8)). No label swap was done for SILAC experiments. Light amino acids were purchased from Sigma and medium and heavy amino acids were from Cambridge isotope Labs. SILAC RPMI was supplemented with 10% dialyzed fetal bovine serum (Gibco), 2 mM L-glutamine (Gibco) and penicillin-streptomycin (100 U/mL and 100 µg/ml, Gibco).

Experimental treatment/stimulation of cell cultures

Cells were cultured in complete medium and starved in serum-free medium for 6 hours before short-term stimulation (10-120 min) with ligands (20-100 ng/ml of FGF-2, IGF-1, PDGF-BB or TGFα (Peprotech)) either individually or in a mixture (referred to as “ligand cocktail”). For long-term stimulation (24-72 hours) cells were kept in 0.5% FBS medium and ligands were replenished every 24 hours. For retinoic acid (RA, Sigma-Aldrich) treatment, cells were incubated with 10 µM RA (in complete medium) for indicated time points. For experiments including chemical inhibition, cells were treated for 48-72 hours with the indicated concentration of inhibitor (U0126 (Cell Signaling Technology), OSI-906, NVP-AEW541, SHP099 or Rebastinib (Selleckchem)). The control condition was treated with a final concentration 0.1% DMSO (Sigma-Aldrich) corresponding to amounts for RA- or inhibitor-treated cells (siRNA transfection of cell cultures is described separately below).

Transfection and RNA Interference

For siRNA-mediated knockdown, SH-SY5Y, NB1 and IMR-32 were transfected with target or control siRNA using Lipofectamine RNAiMAX with OptiMem (both Thermo Fisher Scientific/Invitrogen) according to the instructions of the manufacturer. Double-stranded siRNA oligonucleotides targeting human CBL:
(sequence #1: 5’-CCAGCAGAUUGAUAGCUGUACGUAU-3’;
sequence #2: 5’-GCGGAGAAUCAACUCUGAACGGAAA-3’;
sequence #3: 5’-CCUACCAGGACAUCCAGAAAGCUUU-3’)
and targeting CBLB:
(sequence #1: 5’-UCAUCCCACCCUGUUUCCCUGAAUU-3’;
sequence #2: 5’-GGUCCAUCUUCAGAGAAGAAAUCAA-3’;
sequence #3: 5’-CAUGGGAGAGGGUUAUGCCUUUGAA-3’)
and targeting CDK16:
(sequence: 5’-ACAUCGUUACGCUACAUGAtt-3’)
were acquired from (Thermo Fisher Scientific/Invitrogen). For single protein knockdown, cells were transfected with either individual or mixtures of siRNA sequences (as indicated) and for simultaneous knockdown of both Cbl and Cbl-b, cells were transfected with the targeting sequences #3. Experiments were performed with a final siRNA concentration of 25 nM or 50 nM for CDK16 knockdown, and all assays were carried out 24-72 hours post transfection. As a negative control, cells were transfected with Stealth RNAi siRNA GFP Reporter Control duplex (Thermo Fisher Scientific/Invitrogen) in a final concentration corresponding to target siRNAs (25 nM). The knockdown efficiency of target genes was assessed by Western blotting of cell lysates with specific antibodies against Cbl and Cbl-b or CDK16.

Cell lysis, SDS-PAGE and western blotting

For western blotting, cells were washed with phosphate-buffered saline (PBS) and lysed in radio-immunoprecipitation assay (RIPA) buffer (50 mM Tris-HCl pH7.5, 150 mM NaCl, 1 mM EDTA, 1% NP-40, 0.1% sodium deoxycholate with the addition of 5 mM β-glycerophosphate, 5 mM sodium fluoride, 1 mM sodium orthovanadate and 1 cOmplete Protease Inhibitor Cocktail tablet (Roche/Merck) pr. 10 mL of lysis buffer. Protein concentrations were quantified using the Pierce BCA Protein Assay Kit (ThermoFisher Scientific). Proteins were separated by SDS-PAGE using NuPAGE 4-12% Bis-Tris Protein Gels (Invitrogen/Thermo Fisher Scientific) under denaturing and reducing conditions with MOPS SDS running buffer (Invitrogen/Thermo Fisher Scientific) and transferred onto nitrocellulose membranes (Sigma-Aldrich). Membranes were blocked in phosphate-buffered saline (PBS) and 0.1% Tween-20 with either 5% bovine serum albumin (BSA) (Sigma-Aldrich) or 5% skim milk powder (Sigma-Aldrich), before incubation with primary antibody overnight at 4°C. Membranes were incubated for 1 hour with species-specific horseradish peroxidase (HRP)-conjugated secondary antibody and developed and visualized using Novex ECL Chemiluminescent Substrate Reagent Kit (Invitrogen/Thermo Fisher Scientific) and Amersham Hyperfilm (GE Healthcare). For the detection of multiple proteins on the same membrane, antibodies were removed using a stripping buffer [500 mM glycine, pH2.5] and reprobed with a new primary antibody. The ImageJ software was used to quantify the intensity of the protein bands.

The following antibodies were used in this study: rabbit anti-phospho-Akt (Ser473), rabbit anti-Akt, mouse anti-phosho-ERK1/2 (Thr202/Tyr204), rabbit anti-ERK1/2, rabbi anti-PDGFRβ, rabbit anti-Shp-2, rabbit anti-phospho-IGFR1/INSR (Tyr1131/Tyr1146), rabbit anti-IGF1R, anti rabbit-PCTaire1/CDK16 (Cell Signaling Technology), rabbit anti-Cbl, rabbit anti-Cbl-b (Santa Cruz Biotechnology), rabbit anti-EGFR (Millipore), mouse anti-GAPDH (abcam), mouse anti-Vinculin (Sigma-Aldrich), goat-anti-mouse and goat-anti-rabbit HRP-conjugated secondary antibody (Jackson ImmunoReasearch Laboratories).

Cell viability assay – IC50 determination

Cells were seeded in 96-well plates one day prior to inhibitor treatment. For the experiments, cells were treated with increasing concentrations of inhibitor (0.12 μM to 30 μM Rebastinib), (0.004 μM to 1.11 μM OSI-906) in normal growth medium and the control condition was treated with a corresponding amount of vehicle (0.1% DMSO). A cell viability assay was performed after 48 h (Rebastinib) or 72h (OSI-906) of treatment using the Cell counting kit 8 (Sigma-Aldrich), according to the instructions of the manufacturer. Absorbance was measured at 450 nm using a FLUOstar Omega microplate reader from BMG Labtech. Measurements were performed in quadruplicates (Rebastinib) or three biological (nine technical) replicates for the OSI-906 experiments for each condition. An IC_{50} value, corresponding to a concentration yielding a 50% reduction in cell viability was determined using non-linear regression in GraphPad Prism.

Cellular thermal shift assay

Cells were seeded in 15-cm dishes one day prior to the experiment. For experiments, cells were treated with 1.8 μM of Rebastinib (IC_{50}) or a corresponding amount of DMSO (0.1% DMSO) for 30 min. Cells were collected in PBS, centrifuged and resuspended in PBS with 0.4% NP-40 including protease inhibitors. The cell suspensions were equally distributed into PCR tubes and heated for 4 minutes in a thermocycler (Mastercycler, Eppendorf)) at the indicated temperatures (increasing from 37°C to 67°C) before three consecutive snap freeze-thaw cycles using dry ice. Samples were centrifuged (1 h, 20,000xg) and the supernatant was used for immunoblotting against CDK16.

Quantitative Neurite Outgrowth Assay

For quantitative evaluation of neurite outgrowth, SH-SY5Y cells were seeded in complete medium in black 24-well plates (Ibidi) and treated with siRNAs (targeting Cbl and Cbl-b or CDK16), RA, or stimulated with ligands in 0.5% FBS-containing medium (20-100 ng/ml of FGF-2, IGF-1, PDGF-BB or TGFα). Ligands were replenished every 24 hours. For experiments combining siRNA knockdown with chemical inhibition, cells were treated with the indicated concentration of inhibitor (U0126, SHP099 or

Rebastinib), while control cells were incubated with corresponding amounts of DMSO (0.1%). Evaluation of neurite outgrowth was performed 48-72 hours after initiation of the experiment (stimulation or siRNA transfection with/without inhibitor treatment). Prior to image acquisition, nuclei were stained with Hoechst 33342 (Invitrogen/Thermo Fisher Scientific). Visualization and image acquisition were done by live-cell imaging on a ScanR Inverted Microscope high-content screening station (Olympus) equipped with a Hamamatsu Orca flash 4 camera. Images were acquired using a 20x phase contrast objective. Data was acquired by live cell imaging in a closed environmental control system with a humidified atmosphere, CO_2-levels at 5% and temperature-control set to 37°C. Imaging was done in a fully automated fashion using the ScanR acquisition software (Olympus). For each condition, images were acquired on 16 positions each for six replicates, yielding a total of 96 images per condition. Image analysis was done using an in-house developed tool referred to as 'Neurite Outgrowth Quantification'. The tool was developed using *Mathworks* MATLAB version 2018a. This tool enables the automatic segmentation of neurites to extract a measure for the following features: Number of nuclei, area of nuclei and area of neurites.
For basic visualization of neurite outgrowth images were acquired on a phase-contrast microscope (DM1000, Leica) with a DFC420C camera (Leica).

Sample Preparation for Mass Spectrometry Analysis
For all proteomics analyses, cells were washed in PBS and lysed in boiling guanidine-hydrochloride lysis buffer [6 M guanidine-HCl with 100 mM Tris pH8.5, 5 mM tris(2-carboxyethyl)phosphine (TCEP) and 10 mM chloroacetamide]. Collected cell extracts were heated at 99°C for 10 min followed by sonication. Protein concentrations were determined using the Pierce BCA Protein Assay Kit (ThermoFisher Scientific). Proteins were digested with endoproteinase Lys-C for 2-3 hours and diluted to <2 M guanidine-HCl with 25 mM Tris buffer before digestion with trypsin (Sigma-Aldrich) overnight at 37°C. Enzyme activity was quenched by acidification of the samples with trifluoroacetic acid (TFA) and precipitates were removed by centrifugation at 3000 rpm for 5 min. Peptides were purified and concentrated on reversed-phase C18 Sep-Pak cartridges (Waters) and eluted with 40% acetonitrile (ACN) followed by 60% ACN. The eluate was concentrated and acetonitrile removed using a SpeedVac centrifuge, and the peptide concentration was estimated by measuring absorbance at A280 on a NanoDrop (ThermoFisher Scientific).

SILAC-based deep proteome analysis/Offline High pH reversed phase fractionation
For SILAC-based proteome analysis, tryptic peptides were separated and fractionated by high pH (HpH) reversed-phase fractionation using a Waters XBridge BEH130 C18 3.5 µm 4.6 x 250 mm column on an Ultimate 3000 high-pressure liquid chromatography (HPLC) system (Dionex) essentially as described in Batth et al. (Batth et al., 2014) and Bekker-Jensen et al. (Bekker-Jensen et al., 2017). Fractionation was performed at a flow rate of 1 mL/min while constantly introducing 25 mM ammonium bicarbonate (pH8) at 10%. Peptides were separated using a linear gradient of ACN running from 5% to 35% in 62 min, followed by 5 min of 60% ACN before being ramped to 70% ACN for 3 min. For each sample a total of 46 fractions were collected at 90 s intervals using a Dionex AFC-3000 fraction collector. All fractions were acidified by addition of formic acid (FA) to a final concentration of around 0.1% prior to concentration in a SpeedVac centrifuge. The concentration of peptides in each fraction was estimated by measuring absorbance at A280 on a NanoDrop, allowing 1 µg per fraction to be injected for nano-flow liquid chromatography-tandem mass spectrometry (LC-MS/MS).

TMT-labeling and TiO2-based enrichment of phosphopeptides
Based on the absorbance measurements of SepPak-eluted samples identical amounts of each sample were labeled using TMT10-plex reagents (ThermoFisher Scientific) according to the instructions of the manufacturer. Initially peptide samples were adjusted with ACN to reach a concentration of 50% ACN and with HEPES for a final concentration of approximately 30 mM (pH8). Peptides were labeled for 1 hour using 10 µl TMT-reagent per 100 µg of digested peptide and the reaction was quenched for 15 min with 5% hydroxylamine. Samples were mixed and adjusted with 88% ACN, 12% TFA (to 80% ACN,

12% TFA) for phosphopeptide enrichment with titanium dioxide (TiO2) beads (GL Sciences) essentially as described in (Jersie-Christensen et al., 2016). Briefly, TiO2 beads were incubated in a solution of 2.5-dihydrobenzoic acid (DHB) (20 mg/ml, Sigma-Aldrich) in 80% ACN, 1% TFA for 20 min and the bead suspension was added to the adjusted sample (in 1:2 with the amount of labeled peptide) and incubated with rotation for 30 min. After centrifugation the procedure was repeated with the supernatant for a second round of TiO2-based enrichment. Beads were washed with 80% ACN, 6% TFA followed by 50% ACN, 6% TFA and then 80% ACN, 1% TFA followed by 50% ACN, 1% TFA. The beads were then transferred to C8 StageTips and washed with 10% ACN, 1% TFA followed by 80% ACN, 1% TFA prior to elution with 5% ammonia (NH4OH) and 10% ammonia (NH4OH), 25% ACN. Eluted phosphopeptides were concentrated in a SpeedVac centrifuge prior to fractionation. Microflow HpH reversed-phase fractionation of phosphopeptides was performed on an Ultimate 3000 HPLC system (Dionex) using a Waters Aquity CSH C18 1.7 µM 1 x 150 mm column operating at a constant flow rate of 30 µl/min. The system was equipped with two buffer lines; one containing 5 mM ammonium bicarbonate (pH 8) and one containing 100% ACN. Peptides were separated by a linear gradient of ACN from 5% to 25% over 62.5 min followed by a 4.5 min increase to 60% before being ramped to 70% over 3 min. 12 concatenated fractions were collected per sample. All fractions were acidified by addition of 10% formic acid, SpeedVac'ed to dryness and resolubilized with 80% ACN, 0.1% formic acid. Fractions were then concentrated by SpeedVac and adjusted with 5% ACN, 0.1% TFA prior to MS analysis. TMT proteome samples were handled in a similar manner except for excluding the phosphopeptide enrichment and adding a Sep-Pak purification step in between TMT-labeling and HpH fractionation.

Sample preparation for MS-based analysis of ubiquitylated peptides

SILAC-based analysis of ubiquitylated peptides was performed essentially as described in (Akimov et al., 2018). Initially cells were washed in PBS and lysed in guanidine-hydrochloride lysis buffer [6 M guanidine-HCl with 100 mM Tris pH8.5], then snap-frozen in liquid nitrogen and stored at -80°C. Cell extracts were sonicated and centrifuged at high speed for 30 min, prior to reduction and alkylation for 30 min at room temperature with 5 mM TCEP and 10 mM chloroacetamide. Protein concentrations were determined using the Pierce BCA Protein Assay Kit (ThermoFisher Scientific) and equal amounts of the two SILAC conditions were mixed for each sample. Samples were diluted to a guanidine-HCl concentration <2 M using 25 mM Tris buffer and filtered through 0.45 µm PVDF filters (Millipore) before overnight digestion with Lys-C (added in 1:100 with protein ratio). Peptides were purified using C18 SepPak cartridges (Waters) and the eluate was lyophilized for 24 to 36 hours. The lyophilized peptides were dissolved in 20-25 mL of IP buffer [50 mM MOPS pH7.5, 50 mM NaCl, 10 mM sodium phosphate] containing 0.1% Triton X-100. The peptide solution was centrifuged and filtered through 0.45 µm PVDF filters prior to incubation with UbiSite-conjugated matrix under rotation for 5 hours at 4°C. Beads were washed three times with IP buffer (not containing detergent) and three times with 150 mM NaCl. Immunoprecipitated peptides were eluted by three sequential rounds of 5 minute incubations with 0.1% TFA. Following elution from the beads the pooled eluate was pH-adjusted using 1 M ammonium bicarbonate (final concentration of approximately 25 mM) and the peptides were digested with trypsin overnight at 37°C. Ubiquitylated peptide mixtures were separated by step-wise high-pH reversed phase fractionation using a C18 StageTip (two C18 discs and 0.5 cm of C18 beads (ReProsil-Pur AQ, Dr Maisch)). The StageTip was activated with methanol and washed once with Buffer B [50% ACN, 5 mM ammonium hydroxide] and twice with Buffer A [10 mM ammonium hydroxide pH10]. Samples were adjusted to 10 mM ammonium hydroxide corresponding to pH10 and loaded onto the StageTip followed by two washes with Buffer A. The samples were fractionated into 16 fractions by sequential elutions with 10 mM ammonium hydroxide containing increasing amounts of ACN, using the following ACN gradient: 1.75%, 2.75%, 3.5%, 4%, 5%, 5.5%, 6%, 7%, 8%, 9%, 10.5%, 12%, 14%, 17.5%, 25% and 50%. All fractions were dried in a SpeedVac, resolubilized with 80% ACN, 0.1% formic acid and then concentrated by SpeedVac and adjusted with 5% ACN, 0.1% TFA prior to MS analysis.

Nanoflow liquid chromatography-tandem mass spectrometry (LC-MS/MS)

All samples were analyzed using an Easy nLC1000 or -1200 (ThermoFisher Scientific) coupled to a Q-Exactive HF mass spectrometer (ThermoFisher Scientific) through a nanoelectrospray source as described previously (Kelstrup et al., 2014). Peptides were separated on a 15-cm analytical column (75 μM inner diameter) in-house packed with 1.9 μM reversed-phase C18 beads (ReProsil-Pur AQ, Dr Maisch) and column temperature was maintained at 40°C by an integrated column oven (PRSO-V1, Sonation GmbH). Each peptide fraction was injected using an autosampler and separated in turn using different gradients depending on the type of sample being analyzed. For SILAC-based proteome analysis, a 30 min gradient ranging from 10% to 30% Buffer B over 25 min to 45% B in the following 5 min and using a flow of 350 nL/min was applied. For SILAC-based analysis of ubiquitylated peptides a 60 min gradient ranging from 5% to 30% Buffer B over 50 min to 45% B in the following 10 min and using a flow of 250 nL/min was applied. For TMT-based phosphoproteome and proteome analysis a 60 min gradient ranging from 10% to 30% Buffer B over 50 min to 45% B in the following 10 min and using a flow of 250 nL/min was applied.

The mass spectrometer was operated in data-dependent acquisition mode with spray voltage at 2 kV, heated capillary temperature at 275°C and s-lens radio frequency level at 50%. Dynamic exclusion was set to 30 s and experiments acquired in positive polarity mode. For every full scan the TopN most intense ions were isolated and fragmented by higher-energy collisional dissociation (HCD) with a normalized collision energy of 28% or 33%.

For SILAC-based proteome analysis full scan resolution was set to 60,000 at m/z 200 and the mass range was set to m/z 350-1400. Full scan ion target value was 3E6 with a maximum fill time of 45 ms. For fastest HCD-MS/MS scanning a Top20 method was applied with fragment scan resolution of 15,000 and an ion target value of 1E5 with a maximum fill time of 15 ms. For SILAC-based analysis of ubiquitylated peptides full scan resolution was set to 60,000 at m/z 200 and the mass range was set to m/z 375-1500. Full scan ion target value was 3E6 with a maximum fill time of 25 ms. For HCD-MS/MS scanning a Top10 method was applied with fragment scan resolution of 45,000 and an ion target value of 2E5 with a maximum fill time of 86 ms. For TMT-based phosphoproteome and proteome analysis full scan resolution was set to 120,000 at m/z 200 and the mass range was set to m/z 400-1600. Full scan ion target value was 3E6 with a maximum fill time of 20 ms. For HCD-MS/MS scanning a Top10 method was applied with fragment scan resolution of 60,000 and an ion target value of 2E5 with a maximum fill time of 110 ms.

Processing and analysis of mass spectrometry raw data

Raw MS data files were analyzed using the MaxQuant software version 1.5.3.36, 1.5.8.4 and 1.6.0.17 with the integrated Andromeda search engine (Cox and Mann, 2008; Cox et al., 2011) and searched against a target/decoy version of the human UniProt database, supplemented with commonly observed contaminants. Cysteine carbamidomethylation was searched as a fixed modification, while protein N-terminal acetylation, oxidized methionine, pyroglutamate formation from glutamine and phosphorylation of serine, threonine and tyrosine were searched as variable modifications for all files. Deamidation of asparagine and glutamine was included as extra variable modification. In addition, ubiquitylation of lysines and N-termini (as GG-remnants), were searched as variable modifications for ubiquitin pulldown samples. Maximum peptide mass was set to 7500 Da. A false discovery rate (FDR) of 1% was applied for identifications on peptide, protein and site level.

Bioinformatic data analysis

Analysis of the proteomics data was performed using Perseus software version 1.5.1.12 or 1.5.2.11 (Tyanova et al., 2016). For TMT 10-plex phosphoproteome and proteome analysis, measured intensities were normalized by quantile-based normalization (Bolstad and Irizarry, 2003) followed by median subtraction within rows. For the TMT phosphoproteome (and proteome) data identified phosphorylation sites/proteins were filtered such that they had to be identified in minimum two replicates for at least one of the sample groups and only sites with a localization probability of at least 0.75 (class I sites) were included in the statistical analysis. Significantly regulated sites and proteins were identified

by ANOVA using a permutation-based FDR of 0.05. For SILAC-based analysis of proteome and ubiquitylome MaxQuant normalized ratios were used. SILAC data were filtered such that a protein or site had to be identified in all of the replicates for at least one of the sample groups in order to be retained in the analysis. For SILAC-based proteome analysis, significantly regulated proteins were identified by ANOVA using a permutation-based FDR of 0.001 and presented with all ratios relative to the siGFP control. Significantly regulated ubiquitin sites were identified by a one-sample t-test using a Benjamini-Hochberg corrected FDR of 0.05. To perform statistical testing for the ubiquitin pulldown the total mean of all ubiquitin site ratios were used. All data were filtered for reversed hits and contaminants. Heatmaps were generated based on unsupervised hierarchical clustering using the Perseus software. GO term enrichment analysis and KEGG pathway enrichment analysis were performed using InnateDb (Lynn et al., 2008). Sequence motif analysis was done using the IceLogo software (Colaert et al., 2009). A sequence motif logo plot of +/- 6 amino acids adjacent to the phosphorylated residue was generated using default parameters with $P<0.01$. The functional interaction networks and associated GO terms were obtained using STRING and Cytoscape version 3.7.0 (Franceschini et al., 2013; Shannon et al., 2003). For networks with phosphoproteome data different site ratios were collapsed into one common ratio for proteins with more than one regulated phosphosite. The InstantClue software was used for generation of selected figures (Nolte et al., 2018).

Statistical analysis

For experiments with effects indicated as fold changes relative to control, statistical significance was determined by one-sample t-test or one-way ANOVA. For neurite outgrowth experiments data were filtered for outliers using the ROUT method with default parameters in GraphPad Prism. Significance testing was done using either a student's t-test or one-way ANOVA. Results with P-values <0.05 were deemed as significant.

Supplemental References

Batth, T.S., Francavilla, C., and Olsen, J.V. (2014). Off-line high-pH reversed-phase fractionation for in-depth phosphoproteomics. J. Proteome Res. *13*, 6176–6186.

Bolstad, B.M., and Irizarry, R.A. (2003). A comparison of normalization methods for high density oligonucleotide array data based on variance and bias. *19*, 185–193.

Cox, J., and Mann, M. (2008). MaxQuant enables high peptide identification rates, individualized p.p.b.-range mass accuracies and proteome-wide protein quantification. Nat. Biotechnol. *26*, 1367–1372.

Cox, J., Neuhauser, N., Michalski, A., Scheltema, R.A., Olsen, J.V., and Mann, M. (2011). Andromeda: A peptide search engine integrated into the MaxQuant environment. J. Proteome Res. *10*, 1794–1805.

Franceschini, A., Szklarczyk, D., Frankild, S., Kuhn, M., Simonovic, M., Roth, A., Lin, J., Minguez, P., Bork, P., Von Mering, C., et al. (2013). STRING v9.1: Protein-protein interaction networks, with increased coverage and integration. Nucleic Acids Res. *41*, 808–815.

Jersie-Christensen, R.R., Sultan, A., and Olsen, J.V. (2016). Simple and Reproducible Sample Preparation for Single- Shot Phosphoproteomics with High Sensitivity. In Phospho-Proteomics: Methods and Protocols, Methods in Molecular Biology, pp. 251–260.

Kelstrup, C.D., Jersie-Christensen, R.R., Batth, T.S., Arrey, T.N., Kuehn, A., Kellmann, M., and Olsen, J.V. (2014). Rapid and deep proteomes by faster sequencing on a benchtop quadrupole ultra-high-field orbitrap mass spectrometer. J. Proteome Res. *13*, 6187–6195.

Lynn, D.J., Winsor, G.L., Chan, C., Richard, N., Laird, M.R., Barsky, A., Gardy, J.L., Roche, F.M., Chan, T.H.W., Shah, N., et al. (2008). InnateDB: Facilitating systems-level analyses of the mammalian innate immune response. Mol. Syst. Biol. *4*.

Nolte, H., Macvicar, T.D., Tellkamp, F., and Krüger, M. (2018). Instant Clue : A Software Suite for Interactive Data Visualization and Analysis. 6–13.

Shannon, P., Markiel, A., Ozier, O., Baliga, N.S., Wang, J.T., Ramage, D., Amin, N., Schwikowski, B., and Ideker, T. (2003). Cytoscape: a software environment for integrated models of biomolecular interaction networks. Genome Res. *13*, 2498–2504.

Tyanova, S., Temu, T., Sinitcyn, P., Carlson, A., Hein, M.Y., Geiger, T., Mann, M., and Cox, J. (2016). The Perseus computational platform for comprehensive analysis of (prote)omics data. Nat. Methods *13*, 731-740.
